# Phase IB/II study of alpelisib combined with paclitaxel in patients with *PIK3CA*-altered metastatic or recurrent gastric cancer

**DOI:** 10.1093/oncolo/oyag245

**Published:** 2026-06-26

**Authors:** Ji-Won Kim, Seonghae Yoon, Min-Hee Ryu, Sung Hee Lim, Seung Tae Kim, Tae-Yong Kim, Hye Sook Han, Minkyu Jung, Jin Won Kim, Jee Hyun Kim, Dae Young Zang, Keun-Wook Lee

**Affiliations:** Department of Internal Medicine, Seoul National University Bundang Hospital, Seoul National University College of Medicine, Seongnam 13620, Republic of Korea; Department of Clinical Pharmacology and Therapeutics, Seoul National University Bundang Hospital, Seoul National University College of Medicine, Seongnam 13620, Republic of Korea; Department of Oncology, Asan Medical Center, University of Ulsan College of Medicine, Seoul 05505, Republic of Korea; Division of Hematology Oncology, Department of Medicine, Samsung Medical Center, Sungkyunkwan University School of Medicine, Seoul 06351, Republic of Korea; Division of Hematology Oncology, Department of Medicine, Samsung Medical Center, Sungkyunkwan University School of Medicine, Seoul 06351, Republic of Korea; Department of Internal Medicine, Seoul National University Hospital, Seoul National University College of Medicine, Seoul 03080, Republic of Korea; Department of Internal Medicine, Chungbuk National University Hospital, Chungbuk National University College of Medicine, Cheongju 28644, Republic of Korea; Division of Medical Oncology, Department of Internal Medicine, Yonsei Cancer Center, Yonsei University College of Medicine, Seoul 03722, Republic of Korea; Department of Internal Medicine, Seoul National University Bundang Hospital, Seoul National University College of Medicine, Seongnam 13620, Republic of Korea; Department of Internal Medicine, Seoul National University Bundang Hospital, Seoul National University College of Medicine, Seongnam 13620, Republic of Korea; Department of Internal Medicine, Hallym University Medical Center, Hallym University College of Medicine, Anyang 14068, Republic of Korea; Department of Internal Medicine, Seoul National University Bundang Hospital, Seoul National University College of Medicine, Seongnam 13620, Republic of Korea

**Keywords:** alpelisib, BYL719, PIK3CA, paclitaxel, gastric cancer

## Abstract

**Background:**

This phase IB/II study assessed safety, pharmacokinetics, and efficacy of alpelisib, a selective PI3Kα inhibitor, plus weekly paclitaxel in patients with *PIK3CA*-altered metastatic or recurrent gastric cancer (GC).

**Methods:**

In phase IB, patients with advanced solid tumors received alpelisib 250 mg (dose level [DL] 0) or 300 mg (DL1) once daily plus paclitaxel 70 mg/m^2^ on Days 1, 8, and 15 every 4 weeks to determine maximum tolerated dose (MTD), dose-limiting toxicity (DLT), and the recommended phase II dose (RP2D). In phase II, patients with *PIK3CA*-altered GC received RP2D, and the primary endpoint was 4-month progression-free survival (PFS). Subjects with diabetes or those with risk factors for diabetes who also had impaired glucose tolerance were excluded. Pharmacokinetic sampling was performed in phase IB at cycle 1, Days 1 (initial dose) and 8 (steady state).

**Results:**

In phase IB, MTD was not reached; RP2D was established at DL1. Two patients developed DLTs at DL0 (grade 3 hyperglycemia, diarrhea, and fatigue) and DL1 (grade 3 hyponatremia). Toxicities were consistent with known PI3Kα inhibitor plus taxane combination profiles and were generally manageable. In phase II (*n* = 9), the 4-month PFS rate was 22.2%, and enrollment was discontinued early for futility; no objective responses were observed, and the median PFS was 2.6 months. Alpelisib exposure, assessed by Cmax and AUC0–24, was within expected ranges, and prior gastrectomy was associated with lower exposure.

**Conclusion:**

Alpelisib plus paclitaxel was tolerable and pharmacologically feasible with a defined RP2D but showed limited efficacy in *PIK3CA*-altered GC.

Lessons Learned

- Alpelisib plus weekly paclitaxel combination was tolerable, and the recommended phase II dose was alpelisib 300 mg once daily with paclitaxel 70 mg/m^2^ on Days 1, 8, and 15 every 4 weeks.
- Clinical activity in *PIK3CA*-altered metastatic or recurrent gastric cancer was limited, leading to early discontinuation for futility.
- Prior gastrectomy may reduce alpelisib exposure, underscoring the need to account for exposure variability when developing oral targeted agents for gastric cancer.

## Trial information

**Table:** 

**TRIAL INFORMATION**	
**Disease**	Phase IB: solid tumors Phase II: gastric cancer (GC)
**Stage of Disease/Treatment**	IV
**Prior Therapy**	Phase IB: progression after standard therapy or no established standard therapy Phase II: one prior therapy
**Type of Study**	Phase IB and II (single arm)
**Primary Endpoints**	Phase IB: maximum tolerated dose (MTD), dose-limiting toxicity (DLT), and recommended phase II dose (RP2D) Phase II: 4-month progression-free survival (PFS)
**Secondary Endpoints**	Phase IB: safety, pharmacokinetics (PK), pharmacodynamics (PD), and response rate (RR) by RECIST v1.1 Phase II: overall survival (OS), PFS, RR, disease control rate (DCR), duration of response, and safety
**Additional Details of Endpoints or Study Design** **This was an open-label, phase IB/II clinical trial. Patients were enrolled between December 2020 and February 2024. Phase IB part was a dose-escalation study in adult patients with advanced solid tumors, and a 3 + 3 design was applied in 4 dose levels and was conducted at Seoul National University Bundang Hospital (SNUBH). Dose and schedule of study treatment and definition of dose-limiting toxicity (DLT) in phase IB part are shown in Supplementary Methods. Based on the safety results from phase IB part, patients enrolled in phase II part received the recommended phase II dose (RP2D) of alpelisib and paclitaxel. The dose of alpelisib and paclitaxel was adjusted according to the severity of adverse events (AEs) that occurred. The detailed dose reduction criteria of both drugs are described in Supplementary Methods. The phase II part was a multicenter study conducted at 7 institutions in the Republic of Korea.** **All patients were more than 20 years old with a histologically confirmed cancer diagnosis. All patients had an Eastern Cooperative Oncology Group performance status (ECOG PS) of 0 or 1 with adequate bone marrow, renal, and hepatic function at screening. Patients were excluded if they had symptomatic brain metastases, impaired gastrointestinal function, or gastrointestinal disorders that could significantly alter the absorption of alpelisib, or any other clinically significant disease. In addition, subjects were excluded if they had diabetes, regardless of treatment or symptom, or if they had (1) Korean Diabetes Prediction Score** ^1^ **more than 7 plus impaired glucose tolerance, defined by blood glucose of 140–199 mg/dL after a 2-hour 75 g oral glucose tolerance test, (2) previous history of gestational diabetes, or (3) steroid-induced diabetes. In phase IB part, patients with a histologically confirmed metastatic solid tumor that had progressed after approved therapies or for which there was no effective standard therapy were enrolled. Measurable disease per Response Evaluation Criteria in Solid Tumors (RECIST) version 1.1 was not required, and the existence of an evaluable disease was sufficient for enrollment in phase IB part. In phase II part, patients with histologically confirmed metastatic or recurrent GC (MRGC) who had progressed after first-line fluoropyrimidine-based chemotherapy were included. If the subject received adjuvant chemotherapy after curative gastrectomy and the disease recurred during or within 6 months after adjuvant chemotherapy, the adjuvant chemotherapy was considered first-line treatment. Measurable disease per RECIST 1.1 was required in phase II part. Patients should have tumors with *PIK3CA* gene alterations, such as single-nucleotide variants, small indels, amplification, or structural variations, detected by targeted next-generation sequencing (NGS) tests.** **In phase IB part, a 3 + 3 design was applied, and 6 to 18 DLT-evaluable subjects were expected to be enrolled. In phase II part, Simon’s minimax two-stage design was applied for sample size calculation, with one-sided *α* = 0.05 and *β* = 0.2. The number of patients was calculated assuming that the 4-month PFS rate of the alpelisib plus paclitaxel combination would have *P*_1_ = 60%, compared to *P*_0_ = 35%. These values were based on the 4-month PFS rate of 35% in patients with MRGC receiving paclitaxel monotherapy and 60% in those treated with paclitaxel/ramucirumab combination.** ^2^ **This design requires 26 patients. The target accrual was 18 patients at the first stage; 7 or more patients with non-progressive disease at 4 months were required to proceed to the second stage. Considering a 20% drop-out rate, a total of 31 patients were planned to be enrolled in phase II part. The Kaplan-Meier method was used for survival analysis. The Mann-Whitney U test was employed to compare non-parametric data. All statistical tests were two-sided with significance defined as *P *< .05. All analyses were performed using SPSS for Windows version 23.0 (IBM Corp., Armonk, NY).** **The study protocol was reviewed and approved by the institutional review boards of each hospital. The study was conducted following the tenets of the Declaration of Helsinki and the International Conference on Harmonization Guidelines for Good Clinical Practice. Written informed consent was obtained from all the patients before study enrollment. The trial is registered with ClinicalTrials.gov (NCT04526470).**	

## Drug information

**Table:** 

**DRUG INFORMATION**	
**Drug 1**	
**Generic/Working Name**	Alpelisib (BYL719)
**Company Name**	Novartis (Basel, Switzerland)
**Drug Type**	Small molecule
**Drug Class**	Kinase inhibitor
**Dose**	300 mg once daily (phase II part)
**Route**	Oral
**Schedule of Administration**	Once daily
**Drug 2**	
**Generic/Working Name**	Paclitaxel
**Company Name**	Neotax (JW Pharmaceutical, Seoul, Korea) [Phase IB only] In phase II part, paclitaxel, whether original or generic, was used according to availability at each institution.
**Drug Type**	Small molecule
**Drug Class**	Microtubule inhibitor
**Dose**	70 mg/m^2^ on Days 1, 8, and 15 every 4 weeks
**Route**	Intravenous
**Schedule of Administration**	On days 1, 8, and 15 every 4 weeks

**Table:** 

**DOSE ESCALATION TABLE**				
**Dose Level**	**Dose of Paclitaxel**	**Dose of Alpelisib**	**Number Enrolled**	**Number Evaluable for Toxicity**
**-2**	60 mg/m^2^	200 mg QD	0	0
**-1**	70 mg/m^2^	200 mg QD	0	0
**0**	70 mg/m^2^	250 mg QD	6	6
**1**	70 mg/m^2^	300 mg QD	6	6

## Patient characteristics

**Table:** 

PATIENT CHARACTERISTICS (Phase IB Part)	
**Number of Patients, Male**	10 (83.3%)
**Number of Patients, Female**	2 (16.7%)
**Stage**	IV (100%)
**Age: Median (range)**	68.5 (40–81)
**Number of Prior Systemic Therapies: Median (Range)**	3 (1–6)
**Performance Status: ECOG 0**	5 (41.7%)
**Performance Status: ECOG 1**	7 (58.3%)
**Performance Status: ECOG 2 or above**	0 (0%)
**Cancer types or Histologic Subtypes**	GC: 6 (*PIK3CA* mutant, 4; *PIK3CA* wild type, 2) Colorectal cancer: 2 (*PIK3CA* mutant, 1; *PIK3CA* wild type, 1) Breast cancer: 1 (*PIK3CA* mutant) Endometrial cancer: 1 (*PIK3CA* mutant) Tonsillar cancer: 1 (*PIK3CA* wild type amplification) Submandibular gland cancer: 1 (*PIK3CA* mutant)

## Primary assessment method

**Table:** 

**PRIMARY ASSESSMENT METHOD**	
**Title**	Efficacy of phase IB part
**Number of Patients Screened**	13
**Number of Patients Enrolled**	12
**Number of Patients Evaluable for Toxicity**	12
**Number of Patients Evaluated for Efficacy**	12
**Evaluation Method**	RECIST 1.1
**Outcome Notes** **In phase IB part, 12 patients were enrolled at SNUBH between December 2020 and November 2021. In dose level 0 (alpelisib 250 mg once daily and paclitaxel 70 mg/m^2^), one of three patients experienced DLT (grade 3 hyperglycemia, grade 3 diarrhea, and grade 3 fatigue) (** Tables 1 and 2**). Thus, three additional patients were enrolled, and no additional DLT was observed. In dose level 1 (alpelisib 300 mg once daily and paclitaxel 70 mg/m^2^), of the initial 3 subjects, one experienced DLT of grade 3 hyponatremia (123 mEq/L). The possible cause of hyponatremia was newly developed hydronephrosis due to disease progression and was corrected after ureteral stent placement; however, the investigators could not completely rule out the possibility that the investigational product contributed to the onset or worsening of hyponatremia, and therefore considered it a DLT. Therefore, three additional subjects were enrolled in this cohort, and no additional DLT was found. The MTD was not reached within the prespecified dose levels, and dose level 1 (alpelisib 300 mg once daily and paclitaxel 70 mg/m^2^) was selected as the RP2D.** **The median follow-up time was 51.3 months (95% confidence interval [CI], 3.4–99.2). Seven patients (58.3%) were previously exposed to taxane-based chemotherapy. The RR was 16.7% (2 patients) (** Figure 1, Table 3**). Five patients (41.7%) had stable disease (SD). The DCR was 58.3% (7 patients).** **In dose level 0, one patient with endometrial cancer harboring *PIK3CA* G842S had a confirmed PR. In dose level 1, another patient with *PIK3CA*-amplified (copy number = 6.4) tonsillar cancer had a confirmed PR. Among 4 patients with *PIK3CA* mutant MRGC, no response was observed. The median PFS and OS were 2.4 months (95% CI, 1.9–7.7) and 13.9 months (95% CI, 0.0–28.8), respectively (** Figure 2 **). The 4-month PFS rate was 41.7% ± 14.2%.** **PK analyses were performed only in phase IB part. Serial blood samples were collected during cycle 1 on Days 1 and 8 at pre-dose and at 1, 2, 4, and 6 hours post-dose. In addition, 24-hour trough samples were obtained on Days 2 and 9 before alpelisib administration. Plasma alpelisib concentrations were quantified using a validated liquid chromatography-tandem mass spectrometry (LC–MS/MS) assay.** **Due in part to limited sample size, exploratory exposure-response analyses did not demonstrate statistically significant relationships between alpelisib exposure and clinical outcomes, including overall response, PFS, and DLT. Of note, prior gastrectomy (*n* = 5) appeared to influence systemic exposure: Cmax, ss was significantly lower in patients with total or subtotal gastrectomy (*P* = .030,** Figure 3A**), with a borderline difference in AUC0–24, ss (*P* = .052,** Figure 3B**), suggesting reduced absorption after gastrectomy.**	

**Table 1:** Adverse events, regardless of causality (Phase IB cohort, *N* = 12).

Per person (*n* = 12)	All cycles				28 days			
	All grades	Grade 1	Grade 2	Grade 3	All grades	Grade 1	Grade 2	Grade 3
**Diarrhea**	11 (91.7)	5 (41.7)	4 (33.3)	2 (16.7)	9 (75.0)	4 (33.3)	3 (25.0)	2 (16.7)
**Fatigue**	9 (75.0)	2 (16.7)	6 (50.0)	1 (8.3)	6 (50.0)	2 (16.7)	3 (25.0)	1 (8.3)
**Hyperglycemia**	4 (33.3)	1 (8.3)	2 (16.7)	1 (8.3)	2 (16.7)	1 (8.3)	0	1 (8.3)
**Hepatic infection (liver abscess)**	1 (8.3)	0	0	1 (8.3)	0	0	0	0
**Hyponatremia**	1 (8.3)	0	0	1 (8.3)	1 (8.3)	0	0	1 (8.3)
**Neck wound infection**	1 (8.3)	0	0	1 (8.3)	0	0	0	0
**Anorexia**	6 (50.0)	2 (16.7)	4 (33.3)	0	5 (41.7)	2 (16.7)	3 (25.0)	0
**Peripheral sensory neuropathy**	4 (33.3)	1 (8.3)	3 (25.0)	0	1 (8.3)	1 (8.3)	0	0
**Weight loss**	7 (58.3)	5 (41.7)	2 (16.7)	0	3 (25.0)	3 (25.0)	0	0
**AST/ALT increased**	5 (41.7)	3 (25.0)	2 (16.7)	0	3 (25.0)	1 (8.3)	2 (16.7)	0
**Vomiting**	4 (33.3)	2 (16.7)	2 (16.7)	0	1 (8.3)	0	1 (8.3)	0
**Abdominal pain**	3 (25.0)	1 (8.3)	2 (16.7)	0	3 (25.0)	1 (8.3)	2 (16.7)	0
**Fever**	3 (25.0)	1 (8.3)	2 (16.7)	0	1 (8.3)	0	1 (8.3)	0
**Stomatitis**	3 (25.0)	1 (8.3)	2 (16.7)	0	2 (16.7)	1 (8.3)	1 (8.3)	0
**Hypertension**	2 (16.7)	0	2 (16.7)	0	1 (8.3)	0	1 (8.3)	0
**Nausea**	2 (16.7)	0	2 (16.7)	0	2 (16.7)	0	2 (16.7)	0
**Epistaxis**	1 (8.3)	0	1 (8.3)	0	0	0	0	0
**Kidney dysfunction**	1 (8.3)	0	1 (8.3)	0	1 (8.3)	0	1 (8.3)	0
**Skin eruption**	3 (25.0)	3 (25.0)	0	0	2 (16.7)	2 (16.7)	0	0
**Alopecia**	2 (16.7)	2 (16.7)	0	0	0	0	0	0
**Dizziness**	2 (16.7)	2 (16.7)	0	0	1 (8.3)	1 (8.3)	0	0
**Abdominal indigestion**	1 (8.3)	1 (8.3)	0	0	1 (8.3)	1 (8.3)	0	0
**Cough**	1 (8.3)	1 (8.3)	0	0	1 (8.3)	1 (8.3)	0	0
**Dyspnea**	1 (8.3)	1 (8.3)	0	0	0	0	0	0
**Facial edema**	1 (8.3)	1 (8.3)	0	0	0	0	0	0
**Hemoptysis**	1 (8.3)	1 (8.3)	0	0	1 (8.3)	1 (8.3)	0	0
**Myalgia**	1 (8.3)	1 (8.3)	0	0	1 (8.3)	1 (8.3)	0	0

Data are presented as No. (%). There was no grade 4 adverse event.

Abbreviations: ALT, alanine aminotransferase; AST, aspartate aminotransferase.

**Table 2:** Adverse events, excluding “not related” (Phase IB cohort, *N* = 12).

Per person (*n* = 12)	All cycles				28 days			
	All grades	Grade 1	Grade 2	Grade 3	All grades	Grade 1	Grade 2	Grade 3
**Diarrhea**	11 (91.7)	5 (41.7)	4 (33.3)	2 (16.7)	9 (75.0)	4 (33.3)	3 (25.0)	2 (16.7)
**Fatigue**	9 (75.0)	2 (16.7)	6 (50.0)	1 (8.3)	6 (50.0)	2 (16.7)	3 (25.0)	1 (8.3)
**Hyperglycemia**	4 (33.3)	1 (8.3)	2 (16.7)	1 (8.3)	2 (16.7)	1 (8.3)	0	1 (8.3)
**Hyponatremia**	1 (8.3)	0	0	1 (8.3)	1 (8.3)	0	0	1 (8.3)
**Anorexia**	6 (50.0)	2 (16.7)	4 (33.3)	0	5 (41.7)	2 (16.7)	3 (25.0)	0
**Peripheral sensory neuropathy**	4 (33.3)	1 (8.3)	3 (25.0)	0	1 (8.3)	1 (8.3)	0	0
**Weight loss**	6 (50.0)	4 (33.3)	2 (16.7)	0	3 (25.0)	3 (25.0)	0	0
**AST/ALT increased**	4 (33.3)	2 (16.7)	2 (16.7)	0	2 (16.7)	0	2 (16.7)	0
**Vomiting**	4 (33.3)	2 (16.7)	2 (16.7)	0	1 (8.3)	0	1 (8.3)	0
**Stomatitis**	3 (25.0)	1 (8.3)	2 (16.7)	0	2 (16.7)	1 (8.3)	1 (8.3)	0
**Nausea**	2 (16.7)	0	2 (16.7)	0	2 (16.7)	0	2 (16.7)	0
**Abdominal pain**	1 (8.3)	0	1 (8.3)	0	1 (8.3)	0	1 (8.3)	0
**Hypertension**	1 (8.3)	0	1 (8.3)	0	1 (8.3)	0	1 (8.3)	0
**Kidney dysfunction**	1 (8.3)	0	1 (8.3)	0	1 (8.3)	0	1 (8.3)	0
**Skin eruption**	2 (16.7)	2 (16.7)	0	0	1 (8.3)	1 (8.3)	0	0
**Alopecia**	2 (16.7)	2 (16.7)	0	0	0	0	0	0
**Abdominal indigestion**	1 (8.3)	1 (8.3)	0	0	1 (8.3)	1 (8.3)	0	0
**Facial edema**	1 (8.3)	1 (8.3)	0	0	0	0	0	0
**Myalgia**	1 (8.3)	1 (8.3)	0	0	1 (8.3)	1 (8.3)	0	0

Data are presented as No. (%). There was no grade 4 adverse event.

Abbreviations: ALT, alanine aminotransferase; AST, aspartate aminotransferase.

**Table 3:** Gastrectomy status and mutational profile of patients in phase IB part.

Patient	Diagnosis	Gastrectomy status	Tumor fraction (%)	*PIK3CA* mutation (VAF% or CN)	Co-occurring alterations (VAF% or CN)	Dose-limiting toxicity	Objective response	Target lesion change (%)
**1 (DL0)**	GC	Total gastrectomy	N/A	E726K (N/A), E542V (N/A)	None	No	SD	0
**2 (DL0)**	Endometrial cancer	No	40	G842S (2.14)	*ARID1A* Q878* (34.93) and E2250fs (32.01) *CTNNB1* S37C (28.97) *PTEN* R130G (53.02) and R142W (19.12)	Yes	PR	−48.5
**3 (DL0)**	Breast cancer	No	80	Q546R (83.62)	*TP53* H179R (77.01) *FGFR2* Amp (443.01)	No	PD	−32.8
**4 (DL0)**	Colorectal cancer	No	60	E542K (39.41)	*KRAS* G12D (25.57) *APC* c.835-8A>G (27.46) *CDKN1B* S7fs (37.70) *TP53* R342* (55.08)	No	PD	44.2
**5 (DL0)**	Submandibular gland cancer	No	60	H1047R (22.77)	*TP53* Q165* (28.49)	No	SD	−29
**6 (DL0)**	GC	Total gastrectomy	60	WT	*ARID1A* S103Rfs*11 (11.02) *TP53* R306* (12.05)	No	PD	2.8
**7 (DL1)**	GC	Distal gastrectomy	N/A	E545K (N/A)	None	No	SD	0
**8 (DL1)**	GC	Total gastrectomy	40	E545K (17.64)	*ARID1A* R1721* (20.95) *TP53* C275S (21.17)	Yes	PD	0
**9 (DL1)**	Colorectal cancer	No	15	WT	*APC* S770* (20.16) and E1408* (17.52) *ERBB3* D297Y (37.25) *GATA1* G208R (4.90) *AURKA* Amp (9) *BCL2L1* Amp (8) *BRD4* Amp (7) *CDK6* Amp (9) *DNMT1* Amp (7) *DNMT3B* Amp (8) *ESR1* Amp (6) *JAK3* Amp (7) *MAP2K2* Amp (7) *MAPK3* Amp (10) *NOTCH3* Amp (7) *PIK3CG* Amp (9) *PIK3R2* Amp (7) *SRC* Amp (8) *TOP1* Amp (8)	No	PD	14.1
**10 (DL1)**	GC	Subtotal gastrectomy	N/A	H1047R (N/A)	None	No	SD	−26.2
**11 (DL1)**	Tonsillar cancer	No	60	Amp (6.4)	*MYC* Amp (9.4)	No	PR	−34.3
**12 (DL1)**	GC	Distal gastrectomy	N/A	WT	*MYC* Amp (6.0)	No	SD	14.7

Abbreviations: Amp, amplification; CN, copy number; DL, dose level; GC, gastric cancer; N/A, not available; PD, progressive disease; PR, partial response; SD, stable disease; VAF, variant allele frequency; WT, wild type.

**Figure 1.**
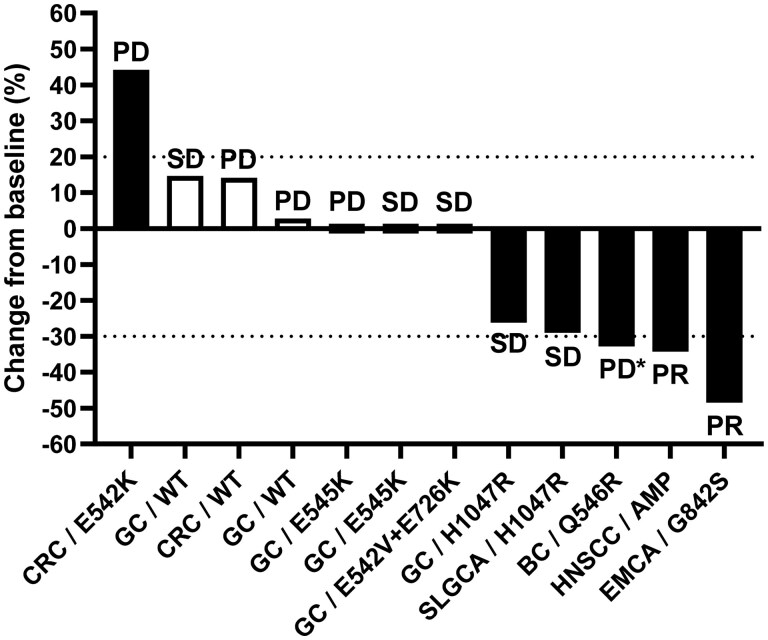
Waterfall plots of the best percentage changes in size of target lesions in phase IB part. *The target lesion response was PR, but new lesions developed in the liver and lung. Abbreviations: BC, breast cancer; CRC, colorectal cancer; EMCA, endometrial cancer; GC, gastric cancer; HNSCC, head and neck squamous cell carcinoma; PD, progressive disease; PR, partial response; SD, stable disease; SLGCA, salivary gland cancer.

**Figure 2.**
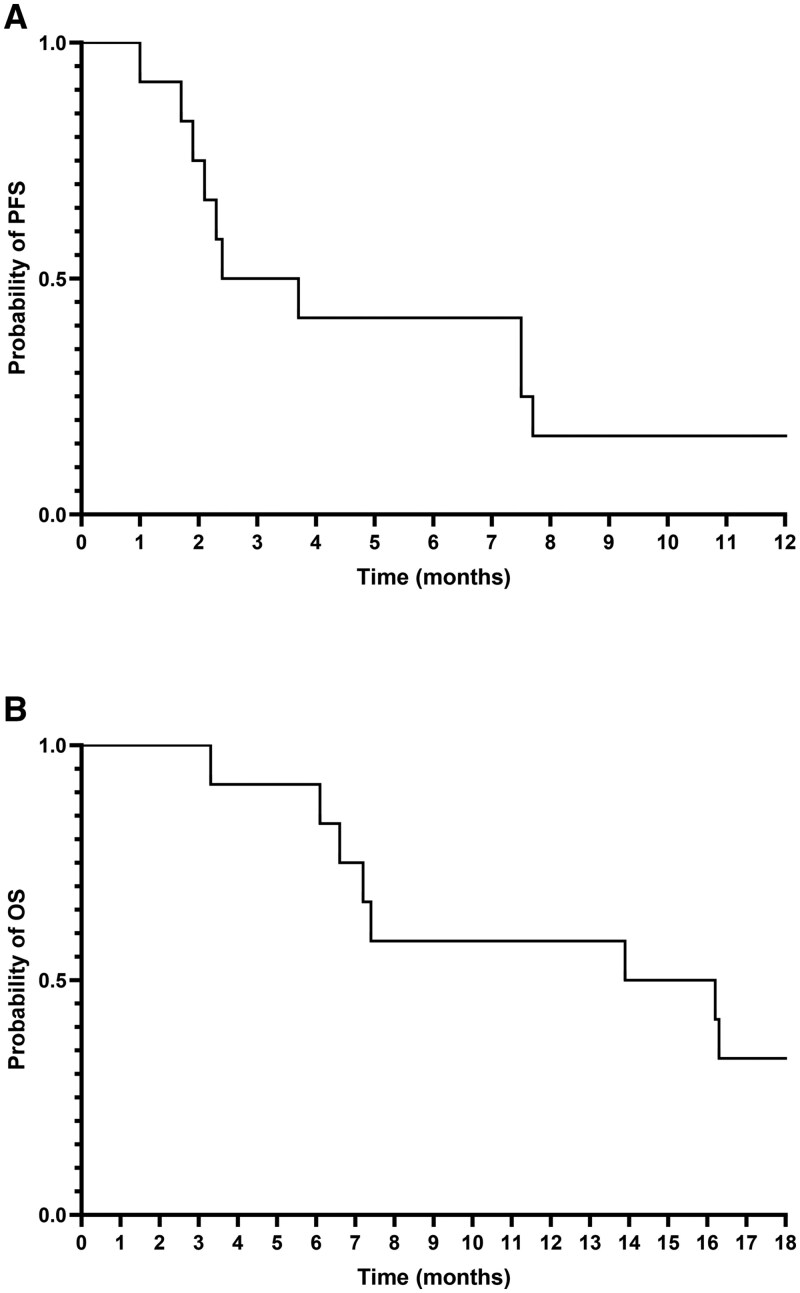
Kaplan-Meier analysis of phase IB cohort (*N* = 12). (A) The median progression-free survival (PFS) was 2.4 months (95% CI, 1.9–7.7 months). The 4-month PFS rate was 41.7% ± 14.2%. (B) The overall survival (OS) was 13.9 months (95% CI, 0.0–28.8 months).

**Figure 3.**
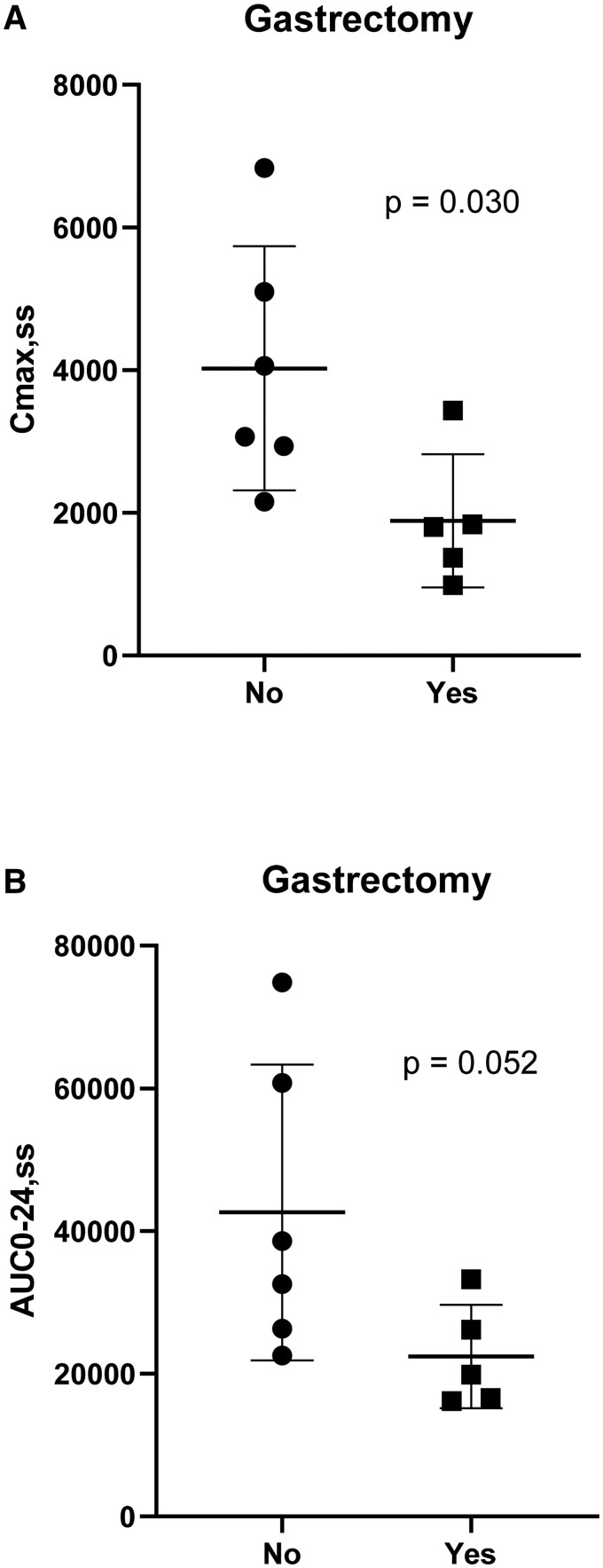
Impact of prior gastrectomy on steady-state alpelisib exposure. (A) Alpelisib Cmax, ss (μg/L) and (B) AUC0–24, ss (h·μg/L) are shown by prior gastrectomy status. Each symbol represents an individual patient, and horizontal bars indicate the mean ± standard deviation. The *P* value reflects between-group comparisons.

## Serious adverse events (SAEs)

**Table:** 

SERIOUS ADVERSE EVENTS		
**Dose Level**	**Adverse Event(s), Grade**	**Attribution**
**0**	Hyperglycemia, Grade 3 Diarrhea, Grade 3 Fatigue, Grade 3	Probable Probable Probable
**1**	Hyponatremia, Grade 3	Possible

## General toxicity profile

See Tables 1 (regardless of causality) and 2 (excluding “not related”).

## Dose limiting toxicities

**Table:** 

DOSE LIMITING TOXICITIES		
**Dose Level**	**Adverse Event(s), Grade**	**Attribution**
**0**	Hyperglycemia, Grade 3 Diarrhea, Grade 3 Fatigue, Grade 3	Probable Probable Probable
**1**	Hyponatremia, Grade 3	Possible

## Pharmacokinetics and pharmacodynamics

**Table:** 

PHARMACOKINETICS/PHARMACODYNAMICS (Cycle 1 Day 1 (single dose))						
**Dose Level**	**Number Enrolled**	**Cmax (μg/L)** **mean ± SD**	**Tmax (h)** **mean (min—max)**	AUC0–24* (h × μg/L) **mean ± SD**	**T½ (h)** **mean ± SD**	**CL/F (L/h)** **mean ± SD**
**0**	6	1,572.6 ± 557.6	2.44 (0.95–3.98)	16,676 ± 5,810	12.19 ± 11.98	13.54 ± 8.03
**1**	6	1,903.9 ± 608.4	3.31 (1.97–5.95)	20,698 ± 6,515	8.84 ± 4.28	13.30 ± 4.80

**Table:** 

PHARMACOKINETICS/PHARMACODYNAMICS (Cycle 1 Day 8 (steady state))						
**Dose Level**	**Number Enrolled**	**Cmax, ss (μg/L)** **mean ± SD**	**Tmax, ss (h)** **mean (min—max)**	AUC0–24, ss* (h × μg/L) **mean ± SD**	**T½ (h)** **mean ± SD**	**CL/F, ss (L/h)** **mean ± SD**
**0**	6	3,682.4 ± 2,073.5	3.26 (1.95–3.95)	40,543 ± 23,029	5.45 ± 0.69	8.21 ± 4.68
**1**	5^†^	2,296.0 ± 978.8	3.81 (2.00–6.03)	24,890 ± 6,228	7.49 ± 3.22	12.75 ± 3.59

* τ = 24 hours.

† One patient could not provide blood samples on cycle 1, Days 8 and 9.

Abbreviations: Cmax, maximum plasma concentration; SD, standard deviation; Tmax, time to maximum plasma concentration; AUC0–24, area under the curve from time 0 to 24 hours; T½, terminal half-life; CL/F, apparent clearance.

## Patient characteristics

**Table:** 

PATIENT CHARACTERISTICS (Phase II Part)	
**Number of Patients, Male**	5 (55.6%)
**Number of Patients, Female**	4 (44.4%)
**Stage**	IV (100%)
**Age: Median (range)**	49 (30–66)
**Number of Prior Systemic Therapies: Median (Range)**	1 (1–1)
**Performance Status: ECOG 0**	3 (33.3%)
**Performance Status: ECOG 1**	6 (66.7%)
**Performance Status: ECOG 2 or above**	0
**Cancer Types or Histologic Subtypes**	GC, 9 (all *PIK3CA* mutant)

## Primary assessment method

**Table:** 

**PRIMARY ASSESSMENT METHOD**	
**Title**	Efficacy of phase II part
**Number of Patients Screened**	11
**Number of Patients Enrolled**	9
**Number of Patients Evaluable for Toxicity**	9
**Number of Patients Evaluated for Efficacy**	9
**Evaluation Method**	RECIST 1.1
**Outcome Notes** **In phase II part, 9 patients were enrolled (** Table 4 **). During a median follow-up time of 8.9 months, the 4-month PFS rate, the primary endpoint, was 22.2% ± 13.9%, and thus further enrollment was stopped due to futility (** Table 5 **). The median PFS and OS were 2.6 months (95% CI, 1.4–3.8) and 3.5 months (95% CI, 0.8–6.2), respectively (** Figure 4 **). The RR and DCR were 0% and 77.8% (7 of 9 patients), respectively (** Figure 5 **). Compared with patients who had undergone gastric surgery (gastrectomy or gastrojejunostomy; *n* = 5), the four patients without prior gastric surgery did not show clearly superior PFS or DCR. However, this comparison was limited by the very small sample size (** Table 6 **).** **The regimen was generally well tolerated (** Table 7 **). Most AEs were grade 1 or 2. Grade 3 or 4 hematologic toxicities were manageable according to the study protocol. In 2 patients (22.2%), grade 4 and 5 gastric hemorrhage events were observed. However, both were assessed by investigators as not related to the study drugs. Other grade 3 AEs were abdominal pain, anorexia, gastric perforation, hyperglycemia, hypoalbuminemia, hypokalemia, pneumonia, skin eruption, and upper respiratory infection (one patient for each). The investigators reported that the two cases of gastric hemorrhage and one case of grade 3 gastric perforation were not related to the investigational product. Two cases of grade 4 anemia resulted from the two cases of gastric hemorrhage. Grade 3 pneumonia and grade 3 upper respiratory infection resulted from an aspiration event and COVID-19 infection, respectively, and thus were not related to the investigational product. Other AEs were manageable.**	

**Table 4:** Patient characteristics (Phase II cohort, *n* = 9).

Clinical characteristics		No. (%)
**Age (years)**	Median (range)	49 (30–66)
**Sex**	Male	5 (55.6)
	Female	4 (44.4)
**ECOG PS**	0	3 (33.3)
	1	6 (66.7)
**Disease status**	Initially metastatic	9 (100)
	Recurrent	0 (0)
**Time to PD on first-line therapy**	<6 months	3 (33.3)
	≥6 months	6 (66.7)
**Tumor grade**	Poorly differentiated	6 (66.7)
	Unknown	3 (33.3)
**Histologic subtype**	Intestinal	1 (11.1)
	Diffuse	2 (22.2)
	Unknown	6 (66.7)
**Previous surgery for gastric cancer**	No	4 (44.4)
	Yes	5 (55.6)
	Palliative total gastrectomy	2 (22.2)
	Palliative gastro-jejunostomy	3 (33.3)
**Site of metastasis**	Peritoneum	5 (55.6)
	Ovary	3 (33.3)
	Bone	2 (22.2)
	Liver	1 (11.1)
	Pleura	1 (11.1)
	Lymph node	1 (11.1)
**Number of metastatic organs**	0 (locally advanced)	2 (22.2)
	1	3 (33.3)
	2	2 (22.2)
	3	2 (22.2)
**Prior therapy for metastatic disease**	Oxaliplatin + fluoropyrimidine-based chemotherapy	8 (88.9)
	XP + trastuzumab	1 (11.1)
**Months from the start of first-line therapy to this clinical trial (median, range)**		12.4 (4.2–44.3)
***PIK3CA* gene alteration**	E545K	3 (33.3)
	E545K + E726K	1 (11.1)
	E542K	1 (11.1)
	H1047R	1 (11.1)
	R93Q	1 (11.1)
	E365K	1 (11.1)
	K111_R115del	1 (11.1)

Abbreviations: ECOG PS, Eastern Cooperative Oncology Group performance status; PD, progressive disease; XP, capecitabine + cisplatin.

**Table 5:** Treatment delivery and outcomes in the phase II cohort (*n* = 9).

Clinical outcomes	No. (%)
**Number of cycles (range)**	2 (1–9)
**Median relative dose intensity, % (range)**	
** Alpelisib**	81.0 (49.3–100)
** Paclitaxel**	78.6 (61.9–100)
**Objective response rate, %**	0
**Disease control rate, %**	77.8
**4-month PFS rate ± SE, %**	22.2 ± 13.9
**Median PFS, months (95% CI)**	2.6 (1.4–3.8)
**Median OS, months (95% CI)**	3.5 (0.8–6.2)

Abbreviations: CI, confidence interval; OS, overall survival; PFS, progression-free survival; SE, standard error.

**Table 6:** Gastrectomy status and mutational profile of patients in phase II part.

Patient	Gastric surgery	Tumor fraction (%)	*PIK3CA* mutation (VAF% or CN)	Co-occurring alterations (VAF% or CN)	Objective response	Target lesion change (%)	Progression-free survival (months)^a^
**1**	Distal gastrectomy	30	R93Q (5.21)	*CASP8* D9Ifs*17 (5.74)	PD	0	1.9
**2**	Gastrojejunostomy	N/A^b^	H1047R (0.24)	None	SD	0	2.6
**3**	Gastrojejunostomy	N/A^b^	E545K (1.17) + E726K (0.41)	*CTNNB1* S37F (0.53) *TP53* R196* (1.12)	SD	−27.7	2.8
**4**	Total gastrectomy	N/A	E545K (14.0)	None	SD	0	8.9
**5**	No	N/A^b^	E545K (0.17)	*ARID1A* Q480* (0.11) and R1721* (0.06)	SD	0	2.2
**6**	No	55	E365K (8.00)	None	SD	−3.3	3.5
**7**	No	25	E545K (1.33)	None	SD	−14	6.1
**8**	Gastrojejunostomy	N/A	E542K (3.56)	*CTNNB1* S45del (44.40) *ERBB2* Amp (65.02) *CCNE1* Amp (12.94)	SD	0	1.0
**9**	No	N/A^b^	K111_R115del (1.27)	*TP53* R248W (1.72)	PD	34.2	1.1

Abbreviations: Amp, amplification; CN, copy number; N/A, not available; PD, progressive disease; SD, stable disease; VAF, variant allele frequency.

a All patients experienced disease progression or death after study treatment.

b ctDNA analysis results.

**Table 7:** Adverse events, regardless of causality (Phase II cohort, *N* = 9).

Per person (*n* = 9)	All grades	Grade 1	Grade 2	Grade 3	Grade 4	Grade 5
**Anemia^a^**	3 (33.3)	0	1 (11.1)	0	2 (22.2)	0
**Neutropenia**	2 (22.2)	0	0	1 (11.1)	1 (11.1)	0
**Gastric hemorrhage^a^**	2 (22.2)	0	0	0	1 (11.1)	1 (11.1)
**Abdominal pain**	5 (55.6)	3 (33.3)	1 (11.1)	1 (11.1)	0	0
**Anorexia**	4 (44.4)	2 (22.2)	1 (11.1)	1 (11.1)	0	0
**Gastric perforation^a^**	1 (11.1)	0	0	1 (11.1)	0	0
**Hyperglycemia**	1 (11.1)	0	0	1 (11.1)	0	0
**Hypoalbuminemia**	1 (11.1)	0	0	1 (11.1)	0	0
**Hypokalemia**	1 (11.1)	0	0	1 (11.1)	0	0
**Pneumonia^b^**	1 (11.1)	0	0	1 (11.1)	0	0
**Skin eruption**	1 (11.1)	0	0	1 (11.1)	0	0
**Upper respiratory infection^c^**	1 (11.1)	0	0	1 (11.1)	0	0
**Stomatitis**	2 (22.2)	1 (11.1)	1 (11.1)	0	0	0
**Nausea**	7 (77.8)	4 (44.4)	3 (33.3)	0	0	0
**Vomiting**	4 (44.4)	1 (11.1)	3 (33.3)	0	0	0
**Infusion-related reaction**	2 (22.2)	0	2 (22.2)	0	0	0
**Diarrhea**	4 (44.4)	3 (33.3)	1 (11.1)	0	0	0
**Peripheral neuropathy**	2 (22.2)	1 (11.1)	1 (11.1)	0	0	0
**Anxiety**	1 (11.1)	0	1 (11.1)	0	0	0
**Constipation**	1 (11.1)	0	1 (11.1)	0	0	0
**Hypocalcemia**	1 (11.1)	0	1 (11.1)	0	0	0
**Hypophosphatemia**	1 (11.1)	0	1 (11.1)	0	0	0
**Hypotension**	1 (11.1)	0	1 (11.1)	0	0	0
**Increased CRP**	1 (11.1)	0	1 (11.1)	0	0	0
**Peripheral pitting edema**	1 (11.1)	0	1 (11.1)	0	0	0
**Dyspepsia**	2 (22.2)	2 (22.2)	0	0	0	0
**Fatigue**	2 (22.2)	2 (22.2)	0	0	0	0
**Urticaria**	2 (22.2)	2 (22.2)	0	0	0	0
**AST/ALT elevation**	1 (11.1)	1 (11.1)	0	0	0	0
**Confusion**	1 (11.1)	1 (11.1)	0	0	0	0
**Eye disorder**	1 (11.1)	1 (11.1)	0	0	0	0
**Fever**	1 (11.1)	1 (11.1)	0	0	0	0
**Gastritis**	1 (11.1)	1 (11.1)	0	0	0	0
**Hand-foot syndrome**	1 (11.1)	1 (11.1)	0	0	0	0
**Hiccup**	1 (11.1)	1 (11.1)	0	0	0	0
**Insomnia**	1 (11.1)	1 (11.1)	0	0	0	0
**Lower extremity edema**	1 (11.1)	1 (11.1)	0	0	0	0
**Palpitation**	1 (11.1)	1 (11.1)	0	0	0	0

Data are presented as No. (%).

Abbreviations: ALT, alanine aminotransferase; AST, aspartate aminotransferase; CRP, C-reactive protein.

a The investigators determined that the two cases of gastric hemorrhage and one case of gastric perforation were not related to the investigational product. Two cases of grade 4 anemia resulted from the two events of gastric hemorrhage.

b One case of grade 3 pneumonia resulted from an aspiration event and thus was not related to the investigational product.

c This event was a COVID-19 infection and not related to the investigational product.

**Figure 4.**
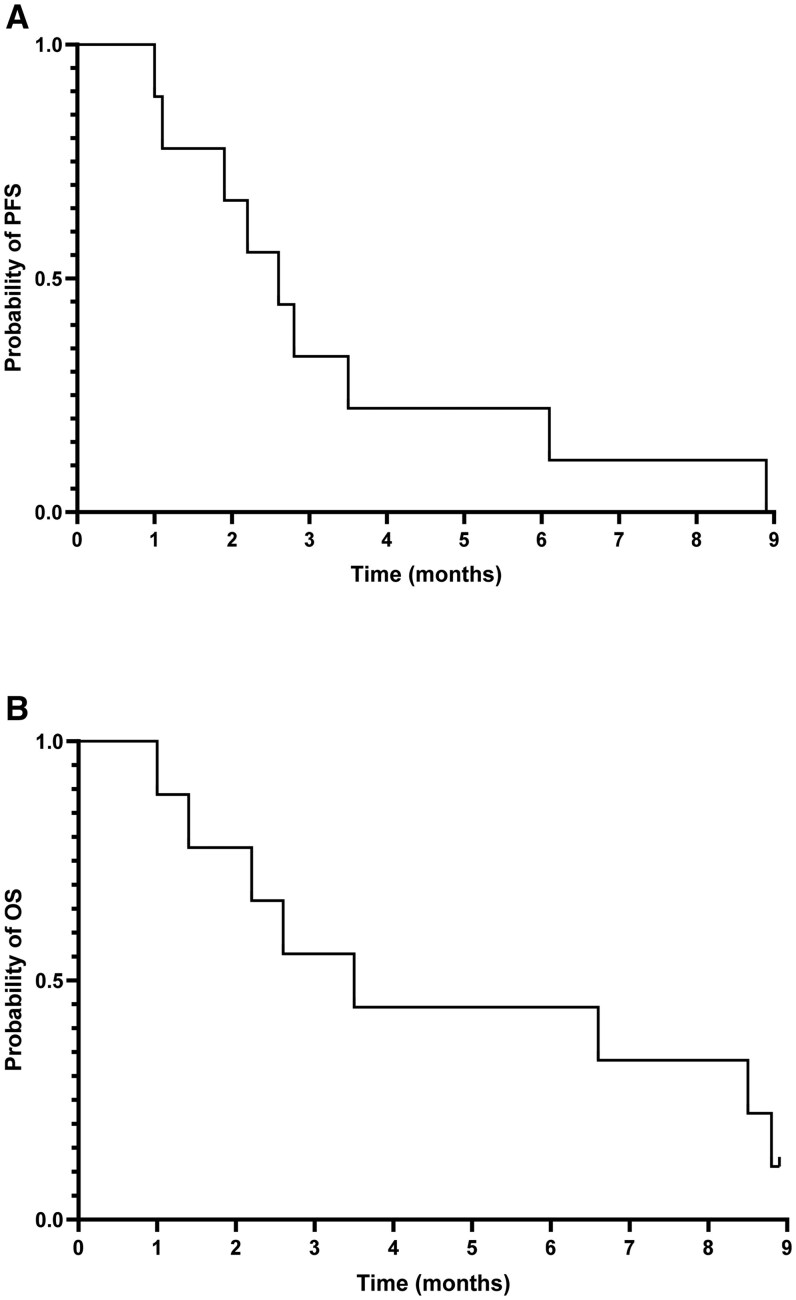
Kaplan-Meier analysis of phase II cohort (*N* = 9). (A) The median progression-free survival (PFS) was 2.6 months (95% CI, 1.4–3.8). The 4-month PFS rate was 22.2% ± 13.9%. (B) The overall survival (OS) was 3.5 months (95% CI, 0.8–6.2 months).

**Figure 5.**
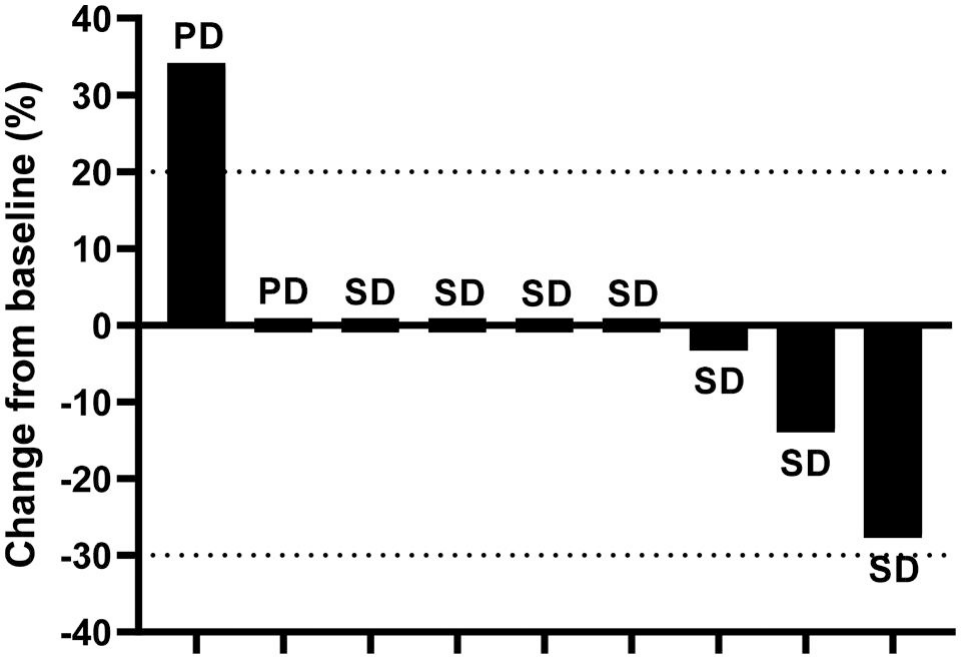
Waterfall plots of the best percentage changes in size of target lesions in phase II part. Abbreviations: SD, stable disease; PD, progressive disease.

## General toxicity profile

See Table 7.

## Discussion

In this phase IB/II study evaluating alpelisib plus paclitaxel in MRGC, the regimen was generally tolerable, but demonstrated limited efficacy in the biomarker-enriched phase II cohort. In phase IB, the RP2D was established as alpelisib 300 mg once daily plus paclitaxel 70 mg/m^2^ on Days 1, 8, and 15 every 4 weeks, with manageable dose-limiting toxicities. In phase II, enrolling patients with *PIK3CA*-altered MRGC, the trial did not meet its prespecified activity threshold: a 4-month PFS rate of 22.2%, no objective responses, and a median PFS of 2.6 months, leading to early closure due to futility.

Safety was consistent with the known profile of PI3Kα inhibition combined with taxane therapy, including hyperglycemia and rash as on-target class effects. In a previous phase IB clinical trial of alpelisib plus paclitaxel in 19 patients with advanced solid tumors, the study was discontinued before RP2D determination due to grade 2 or higher hyperglycemia (*n* = 4) and grade 4 leukopenia (*n* = 1), despite excluding patients requiring insulin therapy or with symptomatic diabetes.^3^ Informed by this experience, our study excluded patients at high risk for diabetes or with predisposing factors such as a history of gestational or steroid-induced diabetes and reduced the paclitaxel dose. As a result, our phase IB study successfully defined RP2D. Current consensus recommendations also emphasize early monitoring and preemptive management to maintain dose intensity.^4^^,^^5^ In our study, most AEs were manageable. Although serious gastrointestinal bleeding and perforation events were observed and adjudicated as unrelated to study treatment by investigators, these events warrant careful monitoring, particularly in patients at high risk for bleeding or perforation.

Our phase IB PK analysis provided additional insights into oral targeted therapy in GC. Alpelisib exposure, as assessed by Cmax and AUC0–24, was within the expected range for 250–300 mg daily dosing, and exploratory exposure–response analyses did not identify statistically significant relationships with efficacy or toxicity, likely in part due to limited sample size and heterogeneity in primary malignancies. Notably, prior gastrectomy was associated with significantly lower Cmax, ss, and a borderline reduction in AUC0–24, ss, suggesting that altered gastrointestinal anatomy may contribute to variability in systemic exposure. Additionally, we attempted an exploratory subgroup analysis of clinical outcomes according to the history of gastric surgery using our phase II cohort; however, this analysis was limited by the small sample size. These gastrectomy findings are hypothesis-generating, but biologically plausible, because oral drug absorption is sensitive to gastric pH, motility, absorptive surface area, and nutrient-dependent dissolution, all of which can be altered after gastric surgery.^6^^,^^7^ Future clinical trials evaluating oral targeted agents in GC should therefore consider gastrectomy status and, where feasible, standardize administration conditions, including meal composition and timing, and incorporate PK-informed approaches to mitigate exposure variability.

The efficacy results of our phase II study should be interpreted in the context of established second-line standards. In the phase III RAINBOW trial,^2^ the addition of ramucirumab to weekly paclitaxel (80 mg/m^2^ on Days 1, 8, and 15 every 4 weeks) prolonged a median PFS from 2.9 to 4.4 months and a median OS from 7.4 to 9.6 months compared with paclitaxel alone; thus, this combination remains a preferred palliative second-line regimen for HER2-negative MRGC. In our study, paclitaxel was administered at a reduced weekly dose (70 mg/m^2^ on Days 1, 8, and 15 every 4 weeks) based on a previous Korean phase III trial (KCSG ST10-01).^8^ In the Korean study, patients receiving weekly paclitaxel at 70 mg/m^2^ achieved a median PFS of 3.5 months and a median OS of 8.6 months; these outcomes appeared comparable to those achieved by the weekly paclitaxel arms (80 mg/m^2^) in the RAINBOW trial (PFS 2.9 months, OS 7.4 months) and the Japanese phase III WJOG4007 trial (PFS 3.6 months, OS 9.5 months).^9^ While a paclitaxel dose of 70 mg/m^2^ was selected for this study based on prior clinical experience and to optimize safety when combined with alpelisib, the possibility cannot be ruled out that this reduced dose, even in combination with alpelisib, may have contributed to the less-than-expected efficacy outcomes. The clinical benefit observed with alpelisib plus paclitaxel appears closer to outcomes historically expected with taxane monotherapy.

Genomic studies have reported that *PIK3CA* alterations occur in approximately 10%–20% of GC and are enriched in Epstein-Barr virus (EBV)-positive or microsatellite instability (MSI)-high/hypermutated tumors.^10^^,^^11^ Our group previously suggested that *PIK3CA*-altered GC may represent a biologically distinct subset that could benefit from tailored therapeutic strategies.^12^ In addition, our preclinical study supported synergy between alpelisib and paclitaxel in *PIK3CA*-mutant GC models.^13^ Nevertheless, our phase II trial did not meet the primary endpoint and was discontinued early due to futility.

These findings highlight the ongoing challenges of PI3K-targeted strategies in solid tumors. A recent review emphasized that adaptive signaling and resistance mechanisms commonly limit the efficacy of PI3K inhibitors.^14^ Co-occurring genomic alterations and tumor-microenvironment interactions may also blunt the effects of selective PI3Kα inhibition, suggesting that more rational combinations to overcome adaptive resistance may be required. For example, in our phase II cohort, patients 3 and 8 harbored co-occurring somatic alterations, including *CTNNB1* alterations and *ERBB2* and *CCNE1* amplification, which may reflect additional oncogenic dependencies or pathway redundancy. In addition, multi-region sequencing studies suggest that a subset of *PIK3CA* mutations can arise later during clonal evolution and may be present only in subclones rather than the entire tumor cell population,^15–17^ potentially limiting the efficacy of PI3Kα inhibitors when the target alteration is not clonal. In our phase II cohort, patients 1, 6, and 7 may harbor subclonal *PIK3CA* mutations, given the tumor purity and variant allele frequency values of cancer panel reports. Because clonality is difficult to infer from targeted sequencing alone, future biomarker strategies may need to incorporate copy-number context, variant allele fraction adjusted for purity, and broader sequencing coverage to better distinguish clonal from subclonal alterations. Moreover, previous preclinical studies suggested that PI3K blockade can rapidly trigger compensatory receptor tyrosine kinase signaling with consequent ERK pathway activation, thereby attenuating the antitumor effects of PI3K inhibition.^18^^,^^19^ Likewise, in our previous preclinical study, *PIK3CA*-mutant GC cells could acquire resistance to alpelisib through PTEN loss with downstream signaling reactivation.^20^ These data underscore the importance of rational combination strategies in *PIK3CA*-mutant GC, rather than PI3Kα inhibition alone.

Our findings are consistent with a phase IB/II study of AZD8186, a PI3Kβ/δ inhibitor, plus paclitaxel in patients with MRGC selected for *PTEN* loss/*PIK3CB* abnormalities.^21^ Both studies demonstrated acceptable tolerability but insufficient efficacy to justify continued enrollment, despite anecdotal benefits in molecularly distinctive cases. Together, these results suggest that while PI3K pathway inhibition can be safely combined with paclitaxel, single-gene enrichment strategies may be inadequate in MRGC, and meaningful benefit may be confined to narrower subgroups.

This study has limitations, including the single-arm design of phase II, which limits comparative assessment against standard therapy. Molecular selection was based on *PIK3CA* mutation status, but detailed genomic subtyping, such as EBV/MSI/chromosomal instability (CIN)/genomically stable (GS), and *PIK3CA* clonality assessment, was limited. Another limitation is that patients with diabetes or at high risk for diabetes with impaired glucose tolerance were excluded to reduce the risk of severe alpelisib-induced hyperglycemia. Although this approach likely contributed to the manageable safety profile observed in this study, it limits the generalizability of the findings to the broader MRGC population, in which diabetes or pre-diabetic metabolic conditions are prevalent.

In summary, alpelisib plus paclitaxel was tolerable and pharmacologically feasible but demonstrated insufficient clinical activity in patients with *PIK3CA*-mutant MRGC. Notably, alpelisib exposure differed by gastrectomy status, highlighting the importance of altered gastrointestinal anatomy when evaluating oral targeted agents in GC. Future strategies, if pursued, should prioritize more stringent biological enrichment, including assessment of clonality and molecular subtype, rational combination partners, and explicit control of exposure variability.

## Supplementary Material

oyag245_Supplementary_Data

## Data Availability

Data is available upon request to the corresponding authors.
