# Supplementary material for: Phase IB/II study of alpelisib combined with paclitaxel in patients with PIK3CA-altered metastatic or recurrent gastric cancer
Source: Oncologist. 2026 Jun 26;31(8):oyag245. doi: 10.1093/oncolo/oyag245 (PMC13379706; doi:10.1093/oncolo/oyag245)
Supplement: oyag245_Supplementary_Data [file oyag245_supplementary_data.docx]

1. Detailed Description for Phase IB

A total of 4 dose levels (Table 2) are planned, and a traditional 3+3 design is applied (Supplementary Figure 1). Three subjects will be enrolled at each dose level, and if no dose-limiting toxicity (DLT) occurs by the completion of one cycle, three subjects will be enrolled at the next dose level. If DLT develops in one out of three subjects, three additional subjects will be enrolled at the same dose level. If ≥2 out of 6 subjects develop DLT, it is considered to exceed the maximum tolerated dose (MTD), and three additional subjects are enrolled in the lower dose level. The recommended phase II dose (RP2D) is determined based on the MTD and toxicity profiles.

If the fasting blood glucose level measured at 2 to 5 days after the first dose is ≥ 126 mg/dL, then metformin should be initiated.


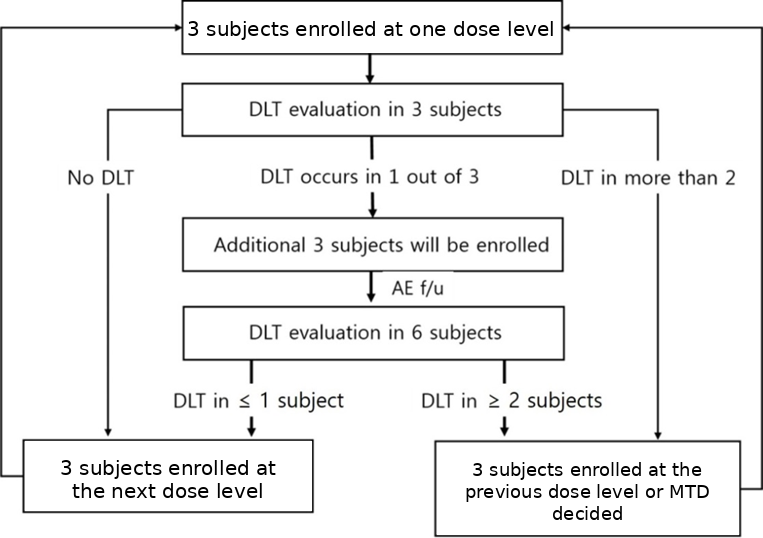


**Supplementary Figure 1. Determination of MTD (3+3 Design)**

**Definition of dose-limiting toxicity (DLT)**

DLT assessment (defined as treatment-related adverse events (AEs) only; DLT does not include AEs associated with the disease [e.g., symptomatic deterioration due to tumor progression]) is only performed during the first cycle and is based on NCI Common Terminology Criteria for Adverse Events (NCI-CTCAE version 4.03) (Supplementary Table 1). The evaluation period of DLT is 28 days (i.e., one cycle treatment period). In the absence of DLT, a subject is considered evaluable for DLT only if paclitaxel is administered all 3 times and alpelisib is administered 75% or more (That is, the completion of taking at least 42 times out of a total of 56 times). Subjects who dropped out of the DLT evaluation in the phase IB part should be replaced with new subjects. DLT is considered in a subject who has received at least one dose of the study treatment, and when any one of the following is developed.

**Supplementary Table 1. Criteria for defining DLTs**

| **TOXICITY** | **DLT CRITERIA** |
| --- | --- |
| Blood and lymphatic system disorders | Febrile neutropenia, Grade ≥ 3  Grade 4 neutropenia for more than 7 consecutive days  Grade 4 thrombocytopenia  Grade 3 thrombocytopenia with bleeding |
| Cardiac disorders | Cardiac toxicity Grade ≥ 3 or cardiac event that is symptomatic or requires medical intervention |
|  | Clinical signs of cardiac disease, such as unstable angina or myocardial infarction, or Troponin Grade 3 (confirmed with a repeat Troponin within 24 h) |
|  | ECG QTc interval prolonged, Grade ≥ 3 |
| Vascular disorders  Hypertension | Persistent hypertension Grade ≥ 3 requiring more than one drug or more intensive therapy than previously administered. |
| General disorders | Fatigue Grade 3 |
| Skin and subcutaneous tissue disorders ^a^:  Rash and/or photosensitivity | Rash or photosensitivity Grade 3 for > 7 consecutive days despite skin toxicity treatment |
|  | Rash or photosensitivity Grade 4 |
| Metabolism and nutrition disorders:  Hyperglycemia  * FPG, fasting plasma glucose | Hyperglycemia Grade 2 (FPG >160 – 250 mg/dL; confirmed with a repeat FPG within 24 h) that does not resolve to grade 1 or less (< 140 mg/dL) within 21 consecutive days (after initiation of oral anti-diabetic treatment  * If insulin is used during grade 2 hyperglycemia, the subject is considered unevaluable for DLT and should be replaced by another subject. |
|  | Hyperglycemia Grade 3 (FPG >250 mg/dL; confirmed with a repeat FPG within 24 h) for > 7 consecutive days despite oral antidiabetic treatment.  * If insulin is used within 7 days after the occurrence of grade 3 hyperglycemia, the subject is considered unevaluable for DLT and should be replaced by another subject. |
|  | Hyperglycemia Grade 4 (FPG >500 mg/dL) |
|  | Hyperglycemia leading to diabetic ketoacidosis, hospitalization for IV insulin  infusion, or non-ketotic coma |
| GI disorders ^a^ | Diarrhea (Grade ≥ 3) ≥ 48 h, despite the use of anti-diarrhea therapy |
|  | Nausea/vomiting (Grade ≥ 3) ≥ 48 h, despite the use of antiemetic therapy |
|  | Pancreatitis Grade ≥ 3 |
| Investigations ^b^  * AST, aspartate aminotransferase  * ALT, alanine aminotransferase  * ANC, absolute neutrophil count | Total bilirubin (blood) Grade 2 for > 7 consecutive days |
|  | Total bilirubin (blood) Grade ≥ 3 |
|  | AST or ALT Grade ≥3 in conjunction with blood bilirubin Grade ≥2 of any duration |
|  | AST or ALT Grade 3 for > 7 consecutive days |
|  | AST or ALT Grade 4 |
|  | Serum alkaline phosphatase Grade 4 for > 7 consecutive days |
|  | Serum creatinine Grade ≥ 3 |
|  | Grade 4 neutropenia (ANC < 500 cells/mm^3^) lasting longer than 7 consecutive days [In the first cycle, the use of G-CSF is not allowed. If G-CSF is administered, the subject is considered unevaluable for DLT and should be replaced with a new subject.] |
|  | Grade 3 thrombocytopenia (Platelet < 50,000/mm^3^) lasting longer than 7 consecutive days |
|  | Grade 3 thrombocytopenia (platelet < 50,000/mm^3^) with bleeding |
|  | Grade 4 thrombocytopenia (platelet < 25,000/mm^3^) |
| Other non-hematologic toxicities | Any other non-hematologic CTCAE Grade ≥3 toxicity that limits the subject's daily life and activities of daily living (ADL) except alopecia. |
| Dose delay of the 2^nd^ cycle | Any other hematologic or non-hematologic toxicity (i.e., greater than at baseline and clinically significant and/or unacceptable) that does not sufficiently respond to supportive care and results in the delay of starting the 2^nd^ cycle more than **14 days**. |
| ^a^ At study initiation, the preemptive use of prophylactic treatment for skin toxicity or nausea/vomiting during Cycle 1 is not planned. However, prophylactic treatment may be initiated in all patients at the dose level where these toxicities have been observed and in all further patients.  Antiemetics may be applied for treatment if the patient has experienced nausea/vomiting CTCAE Grade ≥ 1, at the discretion of the physician.  ^b^ For any CTCAE Grade 3 or 4 hepatic toxicity that does not resolve within 14 days to CTCAE Grade ≤ 1 (or CTCAE Grade ≤ 2 if liver infiltration with tumor present), an abdominal CT scan may be performed to assess if it is related to disease progression.  * Apart from the criteria listed above, if a lower grade AE leads to a dose interruption of more than 7 consecutive days of alpelisib, or 2 or more consecutive doses of paclitaxel within Cycle 1, or between Cycle 1 and Cycle 2 Day 1, this AE will be considered as DLT.  * Paclitaxel (on days 1, 8, and 15) should be administered when ANC ≥ 1,000/mm^3^ and platelet count ≥ 75,000 /mm^3^.  * If Grade 4 neutropenia (ANC < 500/mm^3^) or Grade 3 thrombocytopenia (platelet < 50,000/m^3^) occurs, to determine if the neutropenia and/or thrombocytopenia persists for more than 7 days, a follow-up test should be done on the 8^th^ or later day.  * Note that hypersensitivity reactions are not considered as DLT. However, patients experiencing severe hypersensitivity reactions during cycle 1 despite adequate premedication should be discontinued from the study. | |

1. Dose modifications of study drugs
   1. **Alpelisib**

**2.1.1. General principle of alpelisib dose modification**

In phases IB (after DLT assessment period) and II, dose reduction should be performed according to the alpelisib dose-reduction criteria (Supplementary Tables 2 and 3). If necessary, investigators could decide by consulting the principal investigator in advance, unless it is an emergency that requires immediate action. All dose reductions should be made based on the most severe toxicity previously reported, according to the NCI-CTCAE version 4.03. Once the dose is reduced, dose escalation is not allowed in subsequent cycles. If the treatment is delayed due to toxicity, subsequent visits and examinations should proceed as scheduled, except for drug administration.

Subjects whose dosing schedules are suspended or permanently discontinued due to investigational product (IP)-related AEs should be monitored weekly until the AEs are resolved to grade 1 or lower or stabilized. If the administration is delayed for more than 21 days from the scheduled date, the study treatment should be terminated.

However, if any of the QT-prolonging drugs (Supplementary Table 4) are necessary during the study period, alpelisib should be discontinued during the administration of the QT-prolonging agent. If the subject is to use QT-prolonging medication for a long period and the IP should be discontinued for more than 21 days, the subject should stop the alpelisib treatment permanently.

A missing or omitted dose is not supplemented. It means that the subject does not double the dose even if the previous dose is skipped.

Supplementary Table 2. Dose modification of alpelisib

|  | **Dose level 1** | **Dose level 2** |
| --- | --- | --- |
| Starting dose | 250mg QD | 300mg QD |
| Dose reduction -1 | 200mg QD | 250mg QD |
| Dose reduction -2 | - | 200mg QD |
| Dose reduction should be based on the worst toxicity demonstrated at the last dose. | | |

Supplementary Table 3. Criteria for interruption and re-initiation of alpelisib

| **Adverse drug reaction** | **Dose adjustment and management recommendations** |
| --- | --- |
| **(1) Hematologic toxicities** | |
| **Neutropenia (Absolute neutrophil count [ANC]) or thrombocytopenia** | |
| ANC <0.5 x 10^9^/L without fever and/or platelets < 75 x 10^9^/L | Hold alpelisib until ANC is ≥ 0.5 x 10^9^/L and the platelet count is ≥ 75 x 109/L.  If treatment delay is ≤ 7 days, restart at the same dose  If treatment delay is > 7 days, ↓ 1 dose level |
| ANC <1.0 x 10^9^/L with fever (≥38.3°C) | Hold alpelisib until ANC is ≥ 1.0 x 10^9^/L  If treatment delay is ≤ 7 days, restart at the same dose  If treatment delay is >7 days, ↓ 1 dose level |
| **(2) Hepatic function** | |
| **Bilirubin*** | |
| Grade 1 (>ULN - 1.5 x ULN)  * ULN, upper limit of normal | Maintain dose level with liver function tests (LFTs)* monitored as per protocol |
| Grade 2 (>1.5 - 3.0 x ULN) with ALT or AST ≤ 3.0 x ULN | Omit dose until resolved to ≤ Grade 1, then:  If treatment delay is ≤ 7 days, restart at the same dose  If resolved in > 7 days, ↓ 1 dose level |
| Grade 3 (>3.0 - 10.0 x ULN) with ALT or AST ≤ 3.0 x ULN | Omit dose until resolved to ≤ Grade 1, then:  If treatment delay is ≤ 7 days, ↓ 1 dose level  If resolved in > 7 days, discontinue patient from alpelisib |
| Grade 4 (>10.0 x ULN) | Permanently discontinue alpelisib |
| **AST or ALT** | |
| Grade 1 (>ULN – 3.0 x ULN) | Maintain dose level with LFTs† monitored per protocol |
| Grade 2 (>3.0 - 5.0 x ULN) without total bilirubin elevation to >1.5 x ULN | For patients with grade 0 or 1 at screening  Omit dose until resolved to ≤ baseline value  If treatment delay is ≤ 7 days, restart at the same dose  If resolved in > 7 days, ↓ 1 dose level  For patients with grade 2 at screening  Maintain dose level with LFTs monitored per protocol |
| Grade 3 (>5.0 - 20.0 x ULN) without bilirubin elevation to >1.5 x ULN | Omit dose until resolved to ≤ baseline value, then  If treatment delay is ≤ 7 days, restart at the same dose  If resolved in > 7 days, ↓ 1 dose level |
| Grade 4 (>20.0 x ULN) without bilirubin elevation to >1.5 x ULN | Omit dose until resolved to ≤ baseline value, then ↓ 1 dose level |
| **AST or ALT and concurrent hyperbilirubinemia** | |
| AST or ALT (grade 2: >3.0 – 5.0 x ULN) and total bilirubin (grade 2: >1.5 – 3.0 x ULN) | Omit dose until resolved to ≤grade 1 (bilirubin) and ≤baseline value (AST or ALT), then  If treatment delay is ≤ 7 days, restart at the same dose  If resolved in > 7 days, ↓ 1 dose level |
| AST or ALT (≥grade 3: >5.0 x ULN) and total bilirubin (≥grade 2: >1.5 x ULN) | Permanently discontinue alpelisib |
| * For patients with Gilbert Syndrome, these dose modifications apply to changes in direct bilirubin only  † LFTs include albumin, ALT, AST, total bilirubin (fractionated if total bilirubin > 2.0 x ULN), alkaline phosphatase, and gamma-glutamyl transferase (GGT).  - Patients with grade 0 or 1 at screening experiencing ALT/AST/bilirubin increase ≥ grade 2, the liver function tests must be monitored weekly or more frequently if clinically indicated until resolved to ≤ grade 1.  - In case of any occurrence of ALT/ AST/ bilirubin increase ≥ grade 3, the liver function tests must be monitored weekly or more frequently if clinically indicated until resolved to ≤ grade 1 or baseline value; hereafter, the monitoring should be continued every other week or more frequently if clinically indicated until the end of treatment with study medication.  - Patients who discontinued study treatment should be monitored weekly, including LFTs or more frequently if clinically indicated until resolved to ≤ grade 1 or stabilization (no CTCAE grade change over 4 weeks). | |
| **(3) Hyperglycemia** | |
| Grade 1  (> ULN – 160 mg/dL)  [> ULN - 8.9 mmol/L],  confirmed within 24 hours  * FPG, fasting plasma glucose | Continue alpelisib dosing and maintain the current dose level  - As per the investigator’s discretion, initiate or intensify medication with appropriate anti-diabetic treatment such as oral anti-hyperglycemic therapy (e.g., metformin).  - Check FPG as clinically indicated and at least weekly for 8 weeks, then continue checking at least every 2 weeks. |
| Asymptomatic grade 2  (>160 - 250 mg/dL)  [>8.9 -13.9 mmol/L] | Maintain dose level and re-check within 24 hours: if grade worsens or improves, follow specific recommendations; if grading is confirmed:  - Continue alpelisib dosing.  - Initiate or intensify medication with appropriate anti-diabetic treatment such as oral anti-hyperglycemic therapy (e.g., metformin) as per the investigator’s discretion; consider adding a second oral agent if no improvement after several days.  - Monitor FPG as clinically indicated and at least weekly until FPG resolves to ≤ Grade 1.  - If FPG does not resolve to ≤ Grade 1 within 21 days after institution of appropriate anti-diabetic treatment, reduce alpelisib by 1 dose level.  - Continue with anti-diabetic treatment and check FPG at least weekly for 8 weeks, then continue checking at least every 2 weeks  * If the grade of hyperglycemia deteriorates, treatment according to the deteriorated grade should be conducted. |
| Asymptomatic grade 3  (> 250 - 500 mg/dL)  [> 13.9 - 27.8 mmol/L]  Or Grade 2 with signs or symptoms of hyperglycemia (e.g., mental status  changes, excessive thirst, polyuria) | Omit alpelisib and re-check within 24 hours: if grade worsens or improves, follow specific recommendations. If grading is confirmed:  - Omit alpelisib dosing.  - Consider administering intravenous hydration and intervention for electrolyte/keto-acidosis/hyperosmolar disturbances as clinically appropriate.  - Initiate or intensify medication with appropriate anti-diabetic treatment (consider adding insulin) as per the investigator’s discretion.  - Monitor FPG as clinically indicated and at least twice weekly until FPG resolves to ≤ Grade 1.  - If FPG resolves to Grade 1 within 21 days, then restart alpelisib and reduce 1 dose level  - If FPG doesn’t resolve to Grade 1 within 21 days, then discontinue the patient from alpelisib  - Continue with anti-diabetic treatment and check FPG at least weekly for 8 weeks, then continue checking at least every 2 weeks.  * If the grade of hyperglycemia deteriorates, treatment according to the deteriorated grade should be conducted. |
| Grade 4  (> 500 mg/dL)  [≥ 27.8 mmol/L]  Or Grade 3 with signs or symptoms of hyperglycemia (for ex., mental status  changes, excessive thirst, polyuria) | Omit alpelisib, initiate or intensify medication with appropriate anti-diabetic treatment (consider adding insulin), re-check within 24 hours. If the grade improves, then follow specific grade recommendations. If FPG is confirmed at Grade 4:  - Discontinue the patient from alpelisib.  - Administer intravenous hydration and intervention for electrolyte/ketoacidosis /hyperosmolar disturbances as clinically appropriate.  - Initiate or intensify medication with appropriate anti-diabetic treatment (consider adding insulin) as per the investigator’s discretion  - Check FPG at least weekly for 8 weeks, then continue checking at least every 2 weeks if clinically indicated |
| * Once the grade of hyperglycemia is confirmed through re-measurement of FPG, monitoring of glucose level can then be performed with a self-monitoring blood glucose meter  * A diabetologist consultation should always be considered.  * Based on current experience, hyperglycemia usually resolves within a few days after alpelisib omission; temporary omission of alpelisib may be considered as clinically indicated to improve control of hyperglycemia. Special attention should be paid to the risk of hypoglycemia in patients interrupting alpelisib treatment and receiving insulin or sulfonylurea.  * For all grades: instruct patient to follow dietary guidelines according to local and/or institutional standards for management of diabetes mellitus (such as those provided by the American Diabetes Association) during the study. | |
| **(4) Cardiac** | |
| **Cardiac – Left ventricular systolic dysfunction** | |
| Asymptomatic, resting ejection fraction 40-50%; or 10-20% drop from baseline | Maintain dose level, and continue alpelisib with caution.  Repeat assessment of left ventricular ejection fraction within 4 weeks or as clinically appropriate. |
| Symptomatic, responsive to intervention, ejection fraction 20-39% or > 20% drop from baseline | Omit alpelisib until resolved (patient is asymptomatic, has a resting ejection fraction ≥ 40% and ≤20% decrease from baseline), then ↓ 1 dose level |
| Refractory or poorly controlled, ejection fraction < 20% | Permanently discontinue alpelisib |
| **Cardiac - QTc prolongation** | |
| QTcF ≥ 481ms and ≤ 500 ms | Maintain the dose level of alpelisib and monitor ECG as per the visit schedule.  If the ECG in the additional cycles shows no QTc absolute ≥ 481 ms, then, for subsequent cycles, ECG monitoring will be performed as per the visit schedule.  If the ECG is still abnormal (QTc absolute ≥ 481 msec and ≤500 ms), then ECG monitoring must be performed every 2 weeks until resolution or clinically indicated. |
| QTcF > 500 ms (≥ Grade 3)  or > 60 ms change from baseline on at least two separate ECGs | **- First Occurrence:**  Omit alpelisib.  Perform a repeat ECG within one hour of the first QTcF of > 500 ms or >60ms from baseline: if QTcF remains > 500 ms or >60ms from baseline, repeat ECG as clinically indicated, but at least once a day until the QTcF returns to < 480 ms.  Seek cardiologist input; address electrolytes, calcium, and magnesium abnormalities; concomitant medication must be reviewed.  Once QTcF prolongation has resolved, alpelisib may be restarted at a one lower dose level  - **Second Occurrence:** Permanently discontinue patient from alpelisib. |
| **Cardiac Events (other than QTc prolongation or left ventricular systolic dysfunction)** | |
| Grade 1 or 2 | Maintain dose level |
| Grade 3 | Omit dose until resolved to ≤ Grade 1, then reduce 1 dose level |
| Grade 4 | Permanently discontinue patient from alpelisib |
| **(5) Skin rash** | |
| Grade 1 | Maintain dose level.  - Initiate antihistamine dosing. Recommend non-sedating regimen (e.g., hydroxyzine 25 mg b.i.d) for at least 28 days.  - Topical corticosteroid preparation^a^ for affected areas for at least 28 days. |
| Grade 2 | Maintain the dose level.  - Initiate antihistamine dosing. Recommend a non-sedating regimen during the daytime and sedating at QHS (e.g., hydroxyzine 25 mg AM and noon followed by diphenhydramine 25-50 mg QHS) for at least 28 days.  - Topical corticosteroid preparation^a^ for affected areas for at least 28 days.  - Consider oral corticosteroid (recommend prednisone 0.5-0.75 mg per kg QD or equivalent for 10 days). If rash resolves to Grade 0-1 within 10 days, oral corticosteroid may be discontinued; tapered dosing is not needed. If oral prednisone is administered continuously for >10 days, tapered dosing is indicated. Intravenous steroid administration can be substituted for oral administration.  - If rash is not grade ≤1 in 14 days, administer or continue oral corticosteroid (recommend prednisone 0.5-0.75 mg QD or equivalent for 10 days; longer periods of dosing require tapered dosing). |
| Grade 3/Intolerable grade 2 | Hold alpelisib dosing until rash resolved to Grade 0-1 and consider dermatology consult for skin biopsy and photographs.  - Initiate antihistamine dosing. Recommend a non-sedating regimen during the daytime and sedating at QHS (e.g., hydroxyzine 25 mg a.m. and noon followed by diphenhydramine 25-50 mg QHS) for at least 28 days.  - Topical corticosteroid preparation^a^ for affected areas for at least 28 days.  - Oral corticosteroid (recommend prednisone 0.5-0.75 mg QD or equivalent for 10 days). If rash resolves to Grade 0-1 within 10 days (and does not recur with redosing; see below for guideline on rechallenge), oral corticosteroid may be discontinued; tapered dosing is not needed. If oral prednisone is administered continuously for >10 days, tapered dosing is indicated. Intravenous steroid administration can be substituted for oral administration.  - If rash is not grade ≤1 in 14 days, continue or re-administer oral corticosteroid (recommend prednisone 0.5-0.75 mg QD or equivalent) until resolved.  - Upon rechallenge with alpelisib (once rash Grade ≤1), continue oral corticosteroid for at least 48 hours. If rash and/or pruritus do not recur in 48 hours, discontinue corticosteroid dosing. Antihistamine regimen should be continued for a minimum of 28 days after rechallenge with Alpelisib.  - A dose reduction of one dose level is recommended if this is a second occurrence. Dose reduction is not necessary following the first occurrence of Grade 3 or intolerable Grade 2 rash. |
| Grade 4 | Permanently discontinue patient from Alpelisib and consider a dermatology consult.  Treatment of rash should follow guidelines for Grade 3/intolerable Grade 2 rash above, with the exception of rechallenge and with any additional measures needed. |
| * If steroid is additionally used in subjects who previously had hyperglycemia, check for blood sugar levels and consider adjusting for oral hypoglycemic agents if necessary.  ^a^ Topical corticosteroid preparation recommended regimens:  - For face and/or intertriginous areas (including genitalia), recommend alclometasone 0.05% or hydrocortisone 2.5% creams.  - For other body areas (i.e., trunk and extremities), recommend clobetasol or betamethasone 0.05% creams.  - Consider spray preparation for ease of application on the trunk. For scalp involvement, consider a foam preparation.  Note: Dose of oral corticosteroids given for rash management should be omitted on the day of paclitaxel infusion. Only the dose of corticosteroids given as premedication should be administered. | |
| **(6) Photosensitivity** | |
| Grade 1 | Maintain dose level |
| Grade 2 | Dermatologic consultation is recommended, and prophylactic/therapeutic medication can be used.  Omit dose until resolved to ≤ Grade 1 then:  If resolved in ≤ 7 days, maintain dose level; if photosensitivity of grade 2 redevelops, reduce one dose level.  If resolved in > 7 days, reduce one dose level |
| Grade 3 | Dermatologic consultation is recommended, and prophylactic/therapeutic medication can be used.  Omit dose until resolved to ≤ Grade 1 then:  If resolved in ≤ 7 days, maintain dose level; if photosensitivity of ≥grade 2 redevelops, reduce one dose level.  If resolved in > 7 days, reduce one dose level |
| Grade 4 | Discontinue alpelisib & Dermatologic consultation is recommended. |
| **(7) Eyes disorders** | |
| ≥ Grade 3 ocular/vision symptoms interfering with activities of daily life or requiring medical intervention | Discontinue patient from alpelisib |
| **(8) Stomatitis/Oral mucositis** | |
| Grade 1 / Tolerable Grade 2 | Maintain dose level.  Non-alcoholic or salt water mouthwash. |
| Intolerable Grade 2 or Grade 3 | **First occurrence**: hold until ≤ Grade 1 and ↓ 1 dose level (if stomatitis is readily manageable with optimal management, reintroduction at the same level might be considered at the discretion of the investigator).  **Second occurrence**: hold until ≤ Grade 1 and ↓ 1 dose level. |
| Grade 4 | Permanently discontinue patient from alpelisib. |
| **(9) Diarrhea** | |
| Grade 1 | Maintain dose level |
| Grade 2 | Omit dose until resolved to ≤ Grade 1, then restart at the same dose.  Anti-diarrheal medications, including loperamide should be used as appropriate. |
| Grade ≥ 3 | Omit dose until resolved to ≤ Grade 1, then one dose level reduction can be conducted at the discretion of the investigator.  Anti-diarrheal medications, including loperamide should be used as appropriate. |
| **(10) Pancreatitis** | |
| Grade ≥ 3 | Discontinue alpelisib |
| **(11) Pneumonitis** | |
| Any grade | Immediately interrupt both Alpelisib and paclitaxel for any case of suspected pneumonitis.  Obtain appropriate imaging (high-resolution CT scan) and consider broncho-alveolar lavage (BAL) and biopsy if appropriate based on clinical judgment.  Treatment for pneumonitis should be initiated based on institution guidelines and generally includes high-dose corticosteroids; antibiotic therapy should be administered concurrently if infectious causes are suspected.  Both Alpelisib and paclitaxel should be permanently discontinued in all subjects with confirmed pneumonitis. |
| **(12) Serum creatinine** | |
| < 2 x ULN | Maintain the dose level |
| 2 – 3 x ULN | Omit dose until resolved to ≤ grade 1, then:  If treatment delay is ≤ 7 days, restart at the same dose  If resolved in > 7 days, then ↓ 1 dose level |
| Grade 3 (> 3.0 – 6.0 x ULN) | Permanently discontinue patient from alpelisib |
| Grade 4 ( > 6.0 x ULN) | Permanently discontinue patient from alpelisib |
| **(13) Fatigue (asthenia)** | |
| Grade 1 or 2 | Maintain the dose level |
| Grade 3 | Omit dose until resolved to ≤ Grade 1, then reduce 1 dose level |
| Grade 4 | Permanently discontinue patient from alpelisib |
| **(14) All other adverse events** | |
| Grade 1 or 2 | Maintain the dose level |
| Grade 3 | Omit dose until resolved to ≤ Grade 1, then reduce 1 dose level |
| Grade 4 | Permanently discontinue patient from alpelisib  Note: Omit dose for ≥ Grade 3 vomiting or nausea only if the vomiting or nausea cannot be controlled with optimal antiemetics |

Supplementary Table 4. QT prolonging drugs

| **Drug** | **QT risk(*)** | **Comment** |
| --- | --- | --- |
| Amiodarone | Known risk for TdP | Females>Males, TdP risk regarded as low |
| Arsenic trioxide | Known risk for TdP |  |
| Astemizole | Known risk for TdP | No longer available in the U.S. |
| Bepridil | Known risk for TdP | Females>Males |
| Chloroquine | Known risk for TdP |  |
| Chlorpromazine | Known risk for TdP |  |
| Cisapride | Known risk for TdP | Restricted availability; Females>Males. |
| Disopyramide | Known risk for TdP | Females>Males |
| Dofetilide | Known risk for TdP |  |
| Domperidone | Known risk for TdP | Not available in the U.S. |
| Droperidol | Known risk for TdP |  |
| Halofantrine | Known risk for TdP | Females>Males |
| Haloperidol | Known risk for TdP | When given intravenously or at higher-than-recommended doses, the risk of sudden death, QT prolongation, and torsades increases. |
| Ibutilide | Known risk for TdP | Females>Males |
| Levomethadyl | Known risk for TdP |  |
| Mesoridazine | Known risk for TdP |  |
| Methadone | Known risk for TdP | Females>Males |
| Pentamidine | Known risk for TdP | Females>Males |
| Pimozide | Known risk for TdP | Females>Males |
| Probucol | Known risk for TdP | No longer available in the U.S. |
| Procainamide | Known risk for TdP |  |
| Quetiapine | Possible risk for TdP | Prohibited, as this drug is a sensitive 3A4 substrate |
| Quinidine | Known risk for TdP | Females>Males |
| Sotalol | Known risk for TdP | Females>Males |
| Sparfloxacin | Known risk for TdP |  |
| Tacrolimus | Possible risk for TdP | Prohibited, as this drug is a sensitive 3A4 substrate with a narrow therapeutic index |
| Terfenadine | Known risk for TdP | No longer available in the U.S. |
| Thioridazine | Known risk for TdP |  |
| Vardenafil | Possible risk for TdP | Prohibited, as this drug is a sensitive 3A4 substrate |
| (*) Classification according to the Qtdrugs.org Advisory Board of the Arizona CERT  Sensitive substrates: Drugs whose plasma AUC values have been shown to increase 5-fold or higher when co-administered with a potent inhibitor of the respective enzyme.  **TdP: Torsades de Pointes** | | |

**2.1.2. Management of selected toxicities of alpelisib**

**2.1.2.1. Management of pneumonitis in patients receiving alpelisib**

All patients will be routinely asked about and observed for the occurrence of AEs, including new or changed pulmonary symptoms. Patients who are suspected of having developed pneumonitis should stop study treatment (alpelisib and paclitaxel) immediately and undergo appropriate imaging (CT scan). Broncho-alveolar lavage and biopsy should be considered if clinically appropriate. Infectious causes of lung disease should be ruled out.

Investigators should follow institutional practice for management of pneumonitis, which generally includes treatment with high-dose corticosteroids; antibiotic therapy should be administered concurrently if infectious causes are suspected. Consultation with a pulmonologist is highly recommended for any pneumonitis case during the study treatment. Alpelisib and paclitaxel should be permanently discontinued in all patients with confirmed pneumonitis.

**2.1.2.2. Management of the treatment of skin toxicity in patients receiving alpelisib**

Close monitoring of potential skin reactions will be performed at each planned visit and will be reported as an AE. Rash and hypersensitivity reactions (i.e., maculopapular rash with itching; hypersensitivity reactions defined as rapid recurrence of rash after re-dosing with alpelisib) should be managed with systemic antihistamines and/or low-dose oral or intravenous corticosteroids as detailed in Supplementary Table 3. The use of oral or IV steroids may potentiate hyperglycemia events observed with alpelisib: special attention should be paid to glycemia levels in patients receiving oral or IV steroids.

Although preclinical experiments demonstrated that alpelisib has no potential phototoxic effect, it is recommended to caution patients to avoid sun exposure during treatment with alpelisib, especially when they have already experienced rash or other skin toxicities. Patients should be advised to take measures to protect themselves from direct exposure to sunlight, including the wearing of sunglasses as well as the use of hats, long-sleeve shirts, and long pants when outdoors.

**2.1.2.3. Management of the treatment of alpelisib-induced hyperglycemia**

Alpelisib may affect glucose homeostasis, which could result in increases in plasma glucose levels. Therefore, diabetic patients are excluded from the trial. Patients on treatment for hyperglycemia should always be instructed to follow dietary guidelines provided by the American Diabetes Association. In addition, guidelines for management of hyperglycemia induced by alpelisib include the use of appropriate anti-diabetic treatment, including oral agents or insulin, as detailed in Supplementary Table 3. Patients who develop Grade 3 or 4 hyperglycemia should be managed urgently as per standard clinical practice. Special attention should be paid to the risk of hypoglycemia in patients interrupting alpelisib and receiving insulin or sulfonylurea.

A diabetologist consultation should always be considered. Based on current experience, hyperglycemia usually resolves within a few days after alpelisib omission: temporary omission of alpelisib may be considered as clinically indicated to improve control of hyperglycemia.

- 1. **Paclitaxel**

**2.2.1 General principle of paclitaxel dose modification**

Antihistamine and hydrocortisone (100 mg) will be administered 30 minutes before the administration of paclitaxel to prevent possible hypersensitivity. If hypersensitivity reactions to paclitaxel are not observed, a reduced dose of hydrocortisone to 50 mg is recommended from the following doses to reduce the risk of hyperglycemia. For those who cannot tolerate the previous paclitaxel dose, subsequent dose reductions up to 50 mg/m^2^ are allowed to facilitate the study subjects to continue paclitaxel (Supplementary Table 5). If the RP2D of Paclitaxel is 70 mg/m^2^, it can be reduced up to 50 mg/m^2^ in two steps. If the RP2D is determined to be 60 mg/m^2^, it can be reduced up to 50 mg/m^2^ in one step. Subjects requiring a dose reduction to <50 mg/m^2^ should discontinue paclitaxel treatment.

The criteria for paclitaxel administration on days 1, 8, and 15 of each cycle are summarized in Supplementary Tables 6 and 7. If the AE does not improve to ≤ grade 1 after more than 21 days of discontinuation of the study drugs, the subject should discontinue the study drugs permanently. If a dose reduction has been performed, subsequent dose escalation is not allowed. If one or more of the criteria in the Table are not met at the scheduled treatment period, in case of toxicities or abnormal clinical laboratory values ​​associated with the study drug, the complete blood count and serum chemistry should be repeated once or more per week, and the next dosing schedule should be postponed until the toxicities or abnormal values are recovered. **Even if the cycle is postponed due to toxicity, the radiological evaluation should not be delayed. The radiologic evaluations should be done from the first administration of the study drugs to disease progression, every 8 weeks (± 7 days) within 12 months, and then every 12 weeks (+/- 7 days).**

**Supplementary Table 5. Dose modification of paclitaxel**

| **Paclitaxel** | **Dose level 0** | **Dose level -1** | | **Dose level -2** |
| --- | --- | --- | --- | --- |
|  | 70 mg/m^2^ | 60 mg/m^2^ | | 50 mg/m^2^ |
| Dose reduction should be based on the highest AE rating.  Dose reduction of less than 50 mg/m^2^ is not permitted. | | |  | |

**Supplementary Table 6. Criteria for administering paclitaxel on day 1 in each cycle**

| ANC | ≥ 1.5 x 10^9^/L |
| --- | --- |
| Platelet | ≥ 100 x 10^9^/L |
| Serum Creatinine | ≤ 1 x UNL or calculated creatinine clearance≥ 50mL/min (by Cockcroft-Gault formula, or 24-hour urine collection) |
| Total bilirubin | ≤ 1.5 x UNL |
| AST/ALT | In cases without liver metastases, AST/ALT ≤ 3 x UNL (CTCAE Grade 1)  In cases with liver metastases, ≤ 5 x UNL (CTCAE Grade 2) |
| Paclitaxel-related toxicity/adverse events | NCI-CTCAE version 4.03 ≤ Grade 2 or baseline (alopecia excluded) |

**Supplementary Table 7. Criteria for administering paclitaxel on days 8 and 15 in each cycle**

| ANC | ≥ 1.0 x 10^9^/L |
| --- | --- |
| Platelet | ≥ 75 x 10^9^/L |
| Serum Creatinine | ≤ 1 x UNL or calculated creatinine clearance≥ 50mL/min (by Cockcroft-Gault formula, or 24-hour urine collection) |
| Total bilirubin | ≤ 1.5 x UNL |
| AST/ALT | In cases without liver metastases, AST/ALT ≤ 3 x UNL (CTCAE Grade 1)  In cases with liver metastases, ≤ 5 x UNL (CTCAE Grade 2) |
| Paclitaxel-related toxicity/adverse events | NCI-CTCAE version 4.03 ≤ Grade 2 or baseline (alopecia excluded) |

**2.2.2 Dose reduction and delay of paclitaxel for hematologic toxicities**

If hematologic toxicity occurs, the dose should be reduced or delayed based on the criteria in Supplementary Table 8. Dose reduction within a cycle is not recommended in principle, but it can be done if it is necessary, according to the discretion of the investigators, considering the safety of the subject.

**Supplementary Table 8.** **Dose reduction and delay of paclitaxel for hematologic toxicities**

| **(1) Neutropenia (ANC)** | |
| --- | --- |
| Grade 1 (≥ 1.5 x 10^9^/L) | Maintain the dose level |
| Grade 2 (≥ 1.0 to 1.5 x 10^9^/L) | Maintain the dose level |
| Grade 3 (≥ 0.5 to 1.0 x 10^9^/L) | Hold until ≤ Grade 2.  Resume at the same dose level (May start the next cycle with one level dose reduction at the discretion of the investigator) |
| Grade 4 (<0.5 x 10^9^/L) | Hold until ≤ Grade 2.  Resume paclitaxel with one level dose reduction. |
| **(2) Thrombocytopenia** | |
| Grade 1 (≥ 75 x 10^9^/L) | Maintain the dose level |
| Grade 2 (≥ 50 to 75 x 10^9^/L) | Hold until ≤ Grade 1.  Resume at the same dose level. |
| Grade 3 (≥ 25 to 50 x 10^9^/L) | Hold until ≤ Grade 1.  Resume paclitaxel with one level dose reduction. |
| Grade 4 (<25 x 10^9^/L) | Hold until ≤ Grade 1.  Resume paclitaxel with one level dose reduction. |

**2.2.3 Dose reduction and delay of paclitaxel for non-hematologic toxicities**

If non-hematologic toxicity occurs, it is recommended to adjust the dose or to delay administration based on Supplementary Table 9. In case of nausea or vomiting, it is recommended to administer antiemetics based on the latest version of the NCCN guidelines. In addition, it is recommended to let the subjects know that diarrhea may occur during medication and that they should drink enough fluid. Loperamide may be prescribed and given to the subjects in advance. However, loperamide should not be administered prophylactically. As soon as diarrhea develops, the subjects should take loperamide immediately and drink electrolyte-containing fluids. Study subjects should inform investigators about the symptoms. In addition, loperamide should not be administered for more than 48 hours.

**Supplementary Table 9. Dose reduction and delay of paclitaxel for non-hematologic toxicities**

| **(1) Nausea** | | |
| --- | --- | --- |
| Grade 1 | Maintain the dose level | |
| Grade 2 | Maintain the dose level  May consider holding until ≤ Grade 1.  Resume at the same dose level with appropriate antiemetics. In the 2^nd^ event of nausea (grade 2), despite the use of appropriate antiemetic medication, dose reduction can be conducted. | |
| Grade 3 | Hold until ≤ Grade 1.  Resume at the same dose level with appropriate antiemetics (OR may resume at one level dose reduction at the discretion of the investigator) | |
| Grade 4 | Off paclitaxel | |
| **(2) Vomiting** | | |
| Grade 1 | Maintain the dose level | |
| Grade 2 | Maintain the dose level  May consider holding until ≤ Grade 1.  Resume at the same dose level with appropriate antiemetics. In the 2^nd^ event of vomiting (grade 2), despite the use of appropriate antiemetic medication, dose reduction can be conducted. | |
| Grade 3 | Hold until ≤ Grade 1.  Resume at the same dose level with appropriate antiemetics (OR may resume at one level dose reduction at the discretion of the investigator) | |
| Grade 4 | Off paclitaxel | |
| **(3) Diarrhea/colitis** | | |
| ≤ Grade 1 | Maintain the dose level | |
| Grade 2 | Hold paclitaxel.  Initiate best supportive care (BSC), including loperamide and sufficient fluid intake, and then reassess 24-48 h later. Once ≤ Grade 1 (within 21 days), resume at the same dose level. In the 2^nd^ event of diarrhea/colitis (grade 2), despite the use of appropriate anti-diarrhea medication, dose reduction can be conducted. | |
| Grade 3 | Hold, initiate BSC, and reassess 24-48 h later.  If recovered to ≤ Grade 1 (within 21 days), resume at one level dose reduction if clinically indicated (judged by the investigator) | |
| Grade 4 | Off paclitaxel | |
| Recommended management: Loperamide antidiarrheal therapy  Dosage schedule: 4 mg at first onset, followed by 2 mg with each loose motion until diarrhea-free for 12 hours (maximum dosage: 16 mg/24 hours). Should not be administered for more than 48 consecutive hours.  Anti-diarrheal therapies ancillary to loperamide are also permitted. | | |
| **(4) Peripheral Neuropathy** | | |
| Grade 1 | | Maintain the dose level |
| Grade 2 | | No change in dose or treatment with one level dose reduction, depending on the clinical judgment (may hold the administration of paclitaxel until ≤ Grade 1 at the discretion of the investigator) |
| Grade 3 | | Hold until ≤ Grade 2.  Resume at one level of dose reduction |
| Grade 4 | | Off paclitaxel |
| **(5) Oral Mucositis** | | |
| Grade 1 | | Maintain the dose level |
| Grade 2 | | Hold until ≤ Grade 1.  Resume at the same dose level (May resume at one level dose reduction at the discretion of the investigator) |
| Grade 3 | | Hold until ≤ Grade 1.  Resume at one level of dose reduction |
| Grade 4 | | Off paclitaxel |
| **(6) Other non-hematologic adverse reactions suspicious of relevance** | | |
| Grade 1 | | Maintain the dose level |
| Grade 2 | | Hold until ≤ Grade 1 (May continue paclitaxel administration with BSC).  Resume at the same dose level (May resume at one level dose reduction at the discretion of the investigator). |
| Grade 3 | | Hold until ≤ Grade 1.  Resume at one level of dose reduction |
| Grade 4 | | Off paclitaxel |
